# Push-Pull Zinc Porphyrins as Light-Harvesters for Efficient Dye-Sensitized Solar Cells

**DOI:** 10.3389/fchem.2018.00541

**Published:** 2018-11-16

**Authors:** Jianfeng Lu, Shuangshuang Liu, Mingkui Wang

**Affiliations:** ^1^Wuhan National Laboratory for Optoelectronics, Huazhong University of Science and Technology, Wuhan, China; ^2^School of Chemistry, Monash University, Melbourne, VIC, Australia

**Keywords:** acceptor, donor, porphyrin, push-pull, solar cell

## Abstract

Dye-sensitized solar cell (DSSC) has been attractive to scientific community due to its eco-friendliness, ease of fabrication, and vivid colorful property *etc*. Among various kinds of sensitizers, such as metal-free organic molecules, metal-complex, natural dyes *etc*., porphyrin is one of the most promising sensitizers for DSSC. The first application of porphyrin for sensitization of nanocrystaline TiO_2_ can be traced back to 1993 by using [tetrakis(4-carboxyphenyl) porphyrinato] zinc(II) with an overall conversion efficiency of 2.6%. After 10 years efforts, Officer and Grätzel improved this value to 7.1%. Later in 2009, by constructing porphyrin sensitizer with an arylamine as donor and a benzoic acid as acceptor, Diau and Yeh demonstrated that this donor-acceptor framwork porphyrins could attain remarkable photovoltaic performance. Now the highest efficiencies of DSSC are dominated by donor-acceptor porphyrins, reaching remarkable values around 13.0% with cobalt-based electrolytes. This achievement is largely contributed by the structural development of donor and acceptor groups within push-pull framwork. In this review, we summarized and discussed the developement of donor-acceptor porphyrin sensitizers and their applications in DSSC. A dicussion of the correlation between molecular structure and the spectral and photovoltaic properties is the major target of this review. Deeply dicussion of the substitution group, especially on porphyrin's *meso*-position were presented. Furthermore, the limitations of DSSC for commercialization, such as the long-term stability, sophisticated synthesis procedures for high efficiency dye etc., have also been discussed.

## Introduction

In the global demanding for environmental friendly technologies to achieve efficient light-to-electricity conversion, dye-sensitized solar cells (DSSC) attract world-wide interests due to their specific advantages including high power conversion efficiency (PCE) under weak illumination (Freitag et al., 2017), various colors suitable for building-integrated photovoltaic (Heiniger et al., 2013), low manufacturing cost, compatibility with flexible substrates, possible in hybrid devices such as energy storage *etc* (Hardin et al., 2012; Fakharuddin et al., 2014; Badhulika et al., 2015). Recent studies on water-based electrolytes further offered a strategy to achieve low costs, non-flammability, low volatility and improved environmental compatibility DSSCs (Xiang et al., 2013; Bella et al., 2015; Galliano et al., 2017). Dye-sensitized photoelectrochemical cells are also receiving increasing attention as a promising pathway for visible light-induced water splitting for hydrogen (Fournier et al., 2018). On the other hand, stability test of DSSC based on ionic liquid electrolytes showed a remarkable stability over 2,000 h under full sunlight at 60°C (Marszalek et al., 2014; Cao et al., 2017). One of the major challenges for commercialization of DSSC is the relatively low PCE at standard AM1.5G, which remains around 10 to 14% for decades (Mathew et al., 2014; Kakiage et al., 2015; Lu J. et al., 2018). In fact, there have been extensive efforts to increase the efficiency of DSSC, including development of novel redox couples (Wang et al., 2010, 2012b; Ambre et al., 2012), counter electrodes (Wang et al., 2009; Ku et al., 2013; Wu et al., 2017), nanocrystal semi-conductors (Wang et al., 2012a; Wu et al., 2013; Vittal and Ho, 2017), and new photon-absorbing sensitizers as well (Cao and Wang, 2013; Cho et al., 2018).

**Figure 1:**
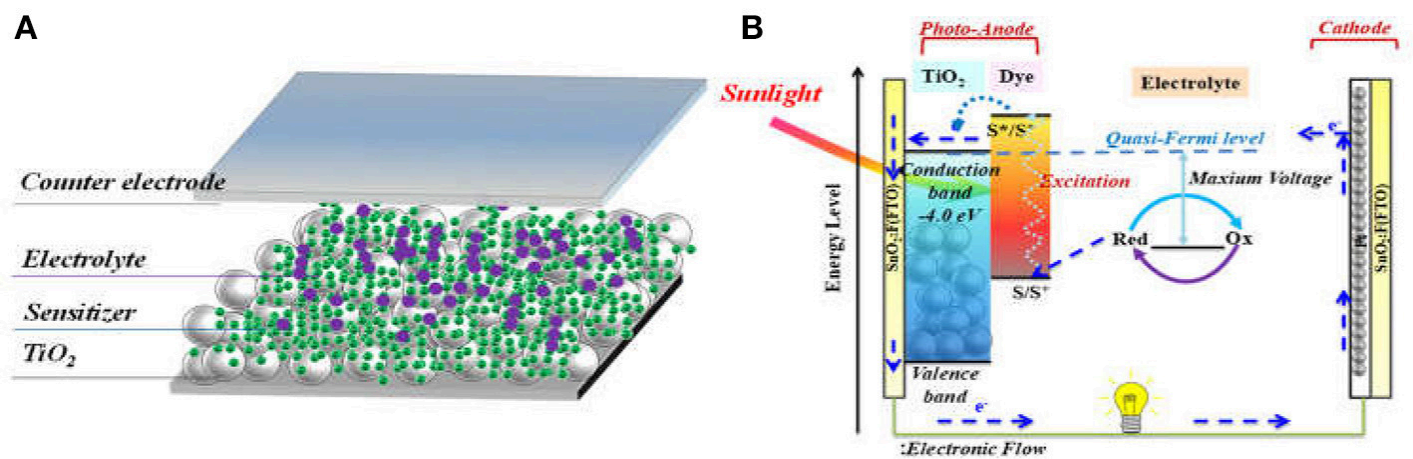
Illustration of typical dye sensitized TiO_2_ solar cell, **(A)** the device structure; **(B)** the working principles.

Mimicking the principles of solar energy conversion of natural photosynthesis, DSSC is often described as “artificial photosynthesis.” Figure 1A shows a typical DSSC structure and Figure 1B illustrates the general working principle of DSSC. It consists of a dye-sensitized nanocrystalline semi-conductor (usually TiO_2_, photo-anode), a counter-electrode (usually Pt-coated fluorine doped tin oxide (FTO) glass, cathode), and electrolyte that contains a redox mediator filling between the two electrodes. After light harvesting by sensitizer, the excited sensitizer injects a hot-electron into the conduction band (CB) of TiO_2_, then the diffusing electrons are collected by the photo-anode FTO, whilst the oxidized dye is regenerated by the redox mediator (usually I^−^/I${}_{3}^{-}$ or [Co(bpy)_3_]^2+/3+^). Finally, the redox couple is regenerated at counter electrode by electron from the photoanode passed through outer load.

In this configuration, the properties of sensitizer, such as their absorption spectrum, energy alignment, function group *etc*. fundamentally determine the photovoltaic performance as well as the color of DSSC (Martin et al., 2016). Historically, ruthenium-polypyridyl complexes have been the most efficient sensitizers, with which the devices exhibited PCE over 10% (Gao et al., 2008; Chen et al., 2009; Cao et al., 2014). The high efficiency can be attributed to the Ru-complex's effectively channeled excitation energy from the metal core to the carboxylate group [i.e., metal to ligand charge transfer (MLCT)]. However, ruthenium complexes suffer from their high cost, rarity and toxicity of Ru, which limit their application in mass-production (Pashaei et al., 2016). Besides Ru-complex, metal-free organic dyes can be prepared with well-established design strategies. Significant progress of organic dyes for DSSC was witnessed in last decade (Ahmad et al., 2013). Nowadays, some research groups reported DSSC with 13% efficiency based on organic dyes (Yao et al., 2015a,b). Nevertheless, narrow absorption region, complicate purification processes, aggregation issues and poor stability of organic dye-based devices need to be solved for future market application (Ye et al., 2015).

**Figure 2:**
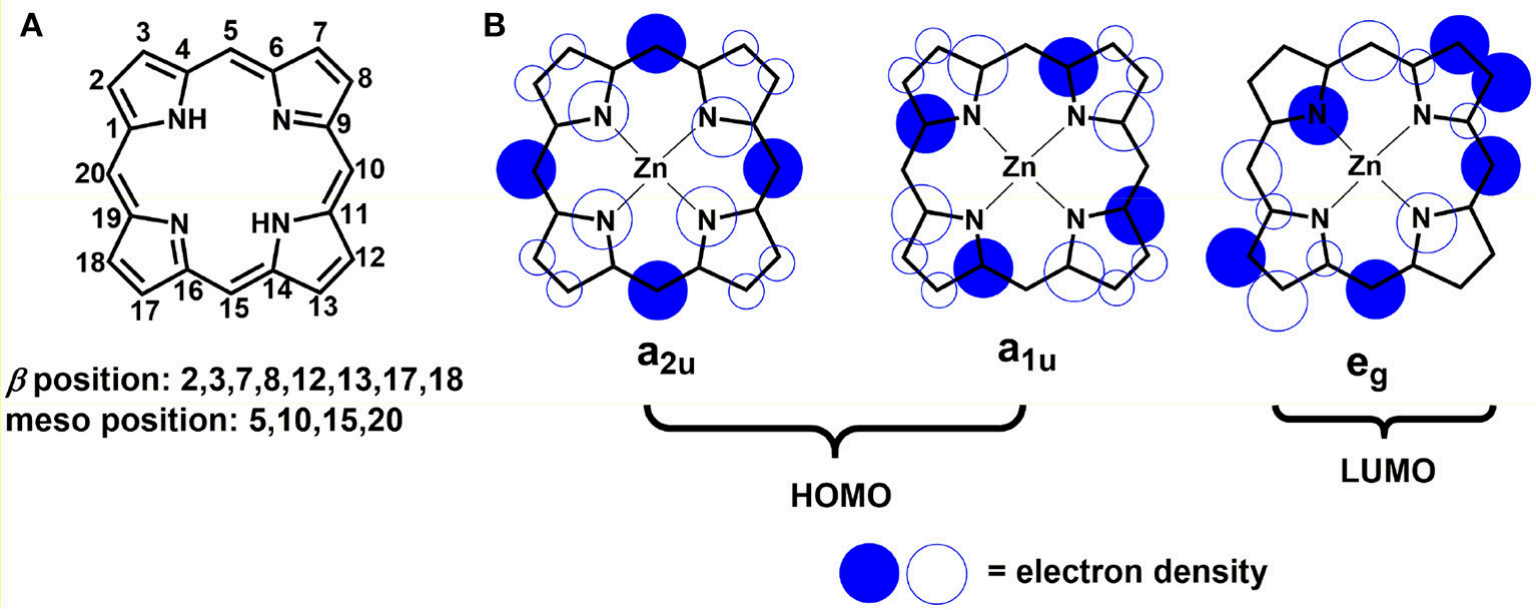
**(A)** The structure and position label of the porphine ring; **(B)** The Gouterman orbital models for the HOMO and LUMOs of porphyrin (re-produced from Sirithip et al., 2015). In the ground state, HOMOs (a_1u_ or a_2u_) have their orbital density mostly on the porphyrin meso positions and the nitrogen atoms with a small amount of electron density on the β-pyrrolic positions; in the excited state, LUMOs (e_g_), which have their electron density on the β-pyrrolic and meso positions.

Given the pivotal role in photosynthesis, the utilization of porphyrins as sensitizer on semi-conductors is of particularly attractive (Ladomenou et al., 2014; Kesters et al., 2015). The word “porphyrin” is derived from a Greek word *porphura* meaning purple, which is how it looks like. Usually, it is a class of deeply colored dye, which has four pyrrole units linked between their α-positions *via* methine bridges (denoted as *meso* atoms). Figure 2A shows a typical porphyrin molecular structure: a planar ring along with an extended π-system. 18 out of 24 π-electrons contribute to the delocalized “conjugation pathway” in the macrocycle. This aromatic characteristic allows the electrophilic substitution reaction on the macrocycle with typical aromatic compounds bearing with halogenation, nitration *etc* (Song et al., 2018). Two different sites on the macrocycle where electrophilic substitution can take place both at *meso* positions of 5, 10, 15, 20, and β positions of 2, 3, 7, 8, 12, 13, 17, and 18 as well (Obraztsov et al., 2017). As established by Gouterman, in the ground state, the orbital density of the highest-occupied molecular orbitals (HOMO) (a_1u_ or a_2u_) mainly distribute on the porphyrin *meso*-positions and the nitrogen atoms; while in the excited state, the orbital density of the lowest-unoccupied molecular orbitals (LUMO) (*e*_*g*_) locate on the β-pyrrolic and *meso* positions (Figure 2B) (Sirithip et al., 2015). Based on this knowledge, one can expect a strong effect on the properties of the macrocyclic core by tailoring its *meso*-positions. As for electronic absorption, most of porphyrins display extreme intense absorption at 400–500 nm (B or Soret band) with molar extinction coefficients over 10^5^ M^−1^ cm^−1^, and moderately intense absorption band at 550–750 nm (Q-band) with 10^4^ M^−1^ cm^−1^. Thus, the absorption bands overlap with the solar radiation across the visible to near-infrared (NIR) region, resulting in panchromatic sensitizers for conversion solar to chemical energy. Therefore, porphyrins have been extensively studied as one of promising components for advanced molecular electronic and photonic devices (Angaridis et al., 2014; Huang et al., 2014).

## The development of porphyrins for solar cell application

**Figure 3:**
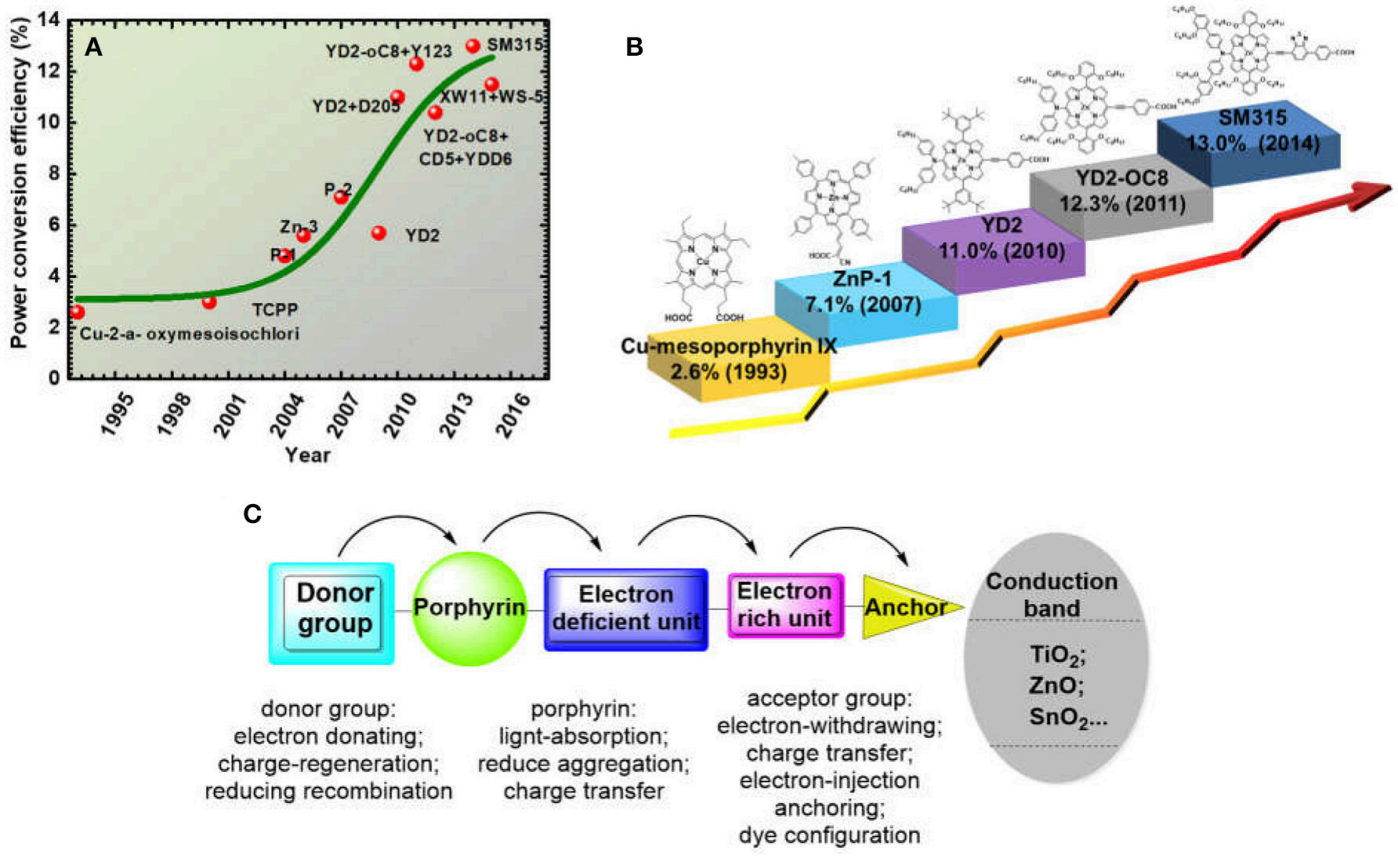
**(A)** The efficiency progressing records of porphyrin based DSSC from the year of 1993 to 2018; **(B)** Some major structure development of porphyrins in DSSC; **(C)** Most popular porphyrin structure with donor-acceptor function groups for recent highly efficient porphyrin dye.

When we look back to the history of porphyrin application in DSSC, the first impressive report is β-substituted chlorophyll derivatives sensitized-TiO_2_ in 1993 (Kay and Graetzel, 1993) (Figure 3). The resulted devices reached a maximum PCE of 2.6%, which is lower than the ruthenium dye at similar conditions because of the ohmic losses at high current densities. After that, the PCE of porphyrin-based DSSC slowly climbed to 3% (Cherian and Wamser, 2000), then to 5.6% (Wang et al., 2005). In 2007, Officer and co-workers reported a β-position functionalised oligomer vinyl electron accepting unit porphyrin ZnP-1 (Figure 3), achieving an overall PCE of 7.1% based on liquid- electrolytes and 3.6% in a solid-state DSSC (ss-DSSC) (Campbell et al., 2007). At that time, the efficiency is relatively low compared with ruthenium sensitizers (~10%). However, this large improvement, especially for thin-film ss-DSSC, encouraged researchers to explore more porphyrin sensitizers for DSSC application.

In 2009, Diau and Yeh reported the first push-pull-structured porphyrin YD2 (Figure 3), achieved an efficiency close to well-known ruthenium dye N719 at similar conditions (Lee et al., 2009). Later, Grätzel and co-workers managed to fill the YD2's absorption gap (480 to 550 nm) between the B and Q band by co-sensitizing with an organic dye, which increased the light-harvesting ability of the devices thus boosted the device PCE up to 11% (Bessho et al., 2010). It is worth to note that this is the first report on ruthenium-free DSSC achieving over 11% efficiency. Diau and Lin firstly reported DSSC based on single porphyrin sensitizer-LD4 with efficiency over 10% (Wang et al., 2011). The efficiency improvement, mainly on the current density, was due to a broadened and red-shifted absorption spectral feature, achieved through a judiciously molecular modification. Another noteworthy porphyrin molecule LD14 was reported in 2011, which also delivered 10% PCE featuring with significantly improved voltage (Chang et al., 2011). Different to YD2 or LD4, the alkyl group in LD14 is on the *meso*-phenyl on the *meta*- position rather than the *ortho*-position. This molecule enveloping strategy successfully protects the metal core from charge recombination with the electrolyte and decreases the molecular aggregation, resulting with 40 mV improvement of the voltage. Based on the above knowledge, Grätzel, Diau, and Yeh designed YD2-o-C8 porphyrin (Figure 3). It remains the diphenylamine (DPA) and benzoic acid as donor and acceptor but with four octyloxy groups attached on the *ortho*-position of the porphyrin ring. The YD2-o-C8 porphyrin successfully delivered a record DSSC PCE of 12.3%, using cobalt-complex redox mediator as electrolyte (Yella et al., 2011). The record was further improved to 13.0% in 2014 with another benchmark porphyrin SM315 (Figure 3), which is similar to the structure of YD2-o-C8 but the conjugation area of donor and acceptor were both extended (Mathew et al., 2014). These results further stimulated the development of porphyrin sensitizer. As we noticed, after 2011, most of the DSSC with high PCE were incorporated with cobalt-complex based electrolyte, suggesting that further DSSC efficiency improvement is not only dependant on the dye structure, but also associated to the advances of the redox couple.

In most highly efficient sensitizers, an efficient inner-charge transfer (ICT) can be attained with an electron withdrawing group (acceptor, A) as one ending side whilst one electron-donating group (donor, D) as another (Chen et al., 2018; Lu J. F. et al., 2018). The electronic interaction between the D and A moieties determines the spectral features as well as electron transfer (Hamann et al., 2008). Generally, the donor and acceptor structure of sensitizer can determine two processes in a working DSSC, oxidized-dye regeneration and hot-electron injection. The donor group is regarded as the dye-regeneration reaction site after electron injection, which should be highly compatible with the redox mediator. In the meantime, the acceptor group plays the role in delivering the electron from the sensitizer to the semi-conductor, accomplishing the electron-hole separation process (O'Regan et al., 2012). Taking the structure of YD2 porphyrin as an example, if considering the porphyrin macrocycle as π-bridge, one can suppose that DPA as donor group to react with I^−^ or [Co(bpy)_3_]^2+^ while 4-ethynylbenzoic acid as acceptor group transferring the electron to semi-conductor. Diau and Lin also suggested that one can envisage porphyrin as a part of electron donor and carboxyl acid as electron acceptor, then the conjugation units between the donor and acceptor as π-bridge. In this case, the whole molecule configuration shows analogous D-π-A character to organic molecules (Wang et al., 2011).

As showed in Figure 3, the first reports on porphyrin application in DSSC are derivatives of chlorophyll and some natural porphyrins, which were not based on design and synthesis. From ZnP-1 (Figure 3B), more concerns were put to the design of porphyrin's molecular structure, such as alkyl chain on porphyrin for the solubility, the length between the porphyrin and anchoring group, the choice of metal core *etc*. The performance of porphyrin based DSSC increased fast from 2009 after Diau and Yeh's report of D-A porphyrin concept (Figure 3C). In the period of 2009 to 2014, a large amount of highly efficient porphyrins were synthesized and reported. After 2011, the rising trend became flat because of the lack of molecular design strategy innovation. Recent advances of porphyrin-based DSSCs and mechanistic studies have been reviewed in several reports (Imahori et al., 2009; Griffith et al., 2012; Li and Diau, 2013; Ladomenou et al., 2014; Urbani et al., 2014; Higashino and Imahori, 2015; Obraztsov et al., 2017). But the analysis on the porphyrin's functional groups is still rare. Generally, a successful molecular engineering is majorly based on a critical construction of the molecular by various substitutions. A single atom replacement in a molecule can lead to an overall change of its properties. In this article, we systematically review the progress of push-pull structured porphyrins based on a rational molecular design approach and their application in DSSC. To clarify the trait of the molecular design ideas, in following sections, we denoted those functional groups at *meso*-positions, 20-position (in Figure 2) as electron-donor, the *para meso*-position (10- position in Figure 2) as acceptor. We focused the discussion on the influence of donor and acceptor structure to the whole molecule's spectral and photovoltaic properties and tried to give strategies for controlling those properties by a rational tailoring of the structure. We also suggested some promising molecular design strategies in the last part of this review, to provide some ideas for further performance improvement.

### Electron-donating groups

**Figure 4:**
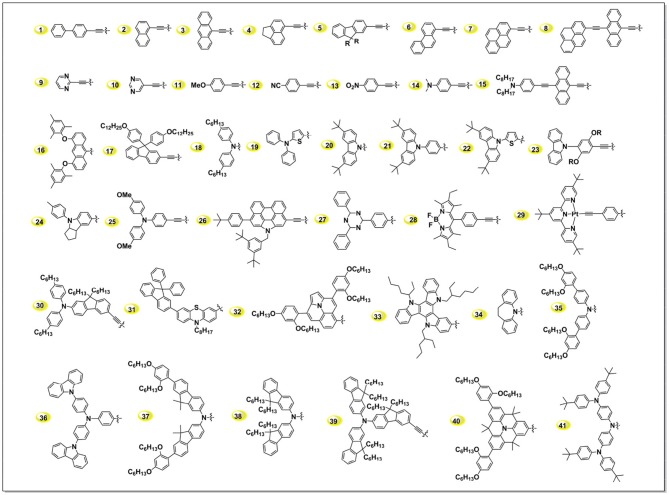
A summary of some donor groups in D-A porphyrins.

In Figure 4, we summarized some typical donor groups reported in last few years. When we take a look at these groups, it is noticed there is a large amount *N*-contained conjugated groups, such as arylamine, alykyamine, carbazole *etc*. Theoretical calculations demonstrated that there exists a driving force from this class of groups pushing the electron-distribution from the dye toward to the TiO_2_, which is benefit to hot-electron injection (Zhang J. et al., 2014). Meanwhile, such *N*-contained groups, for example arylamine, were demonstrated to be able to repel the oxidant in electrolyte from the TiO_2_ surface, which reduces the charge recombination and thus increases the voltage (Barea et al., 2011). Benzene and derivatives such as fluorene, pyrene constructed sensitizers rank at the second position of amount, which usually use to extend the conjugation area of porphyrin molecules thus broaden the light-harvesting area of the device. Hereafter we illustrate several typical donor groups to scrutinize the route for designing highly efficient porphyrin sensitizers.

#### Diarylamino as electron-donating group

**Figure 5:**
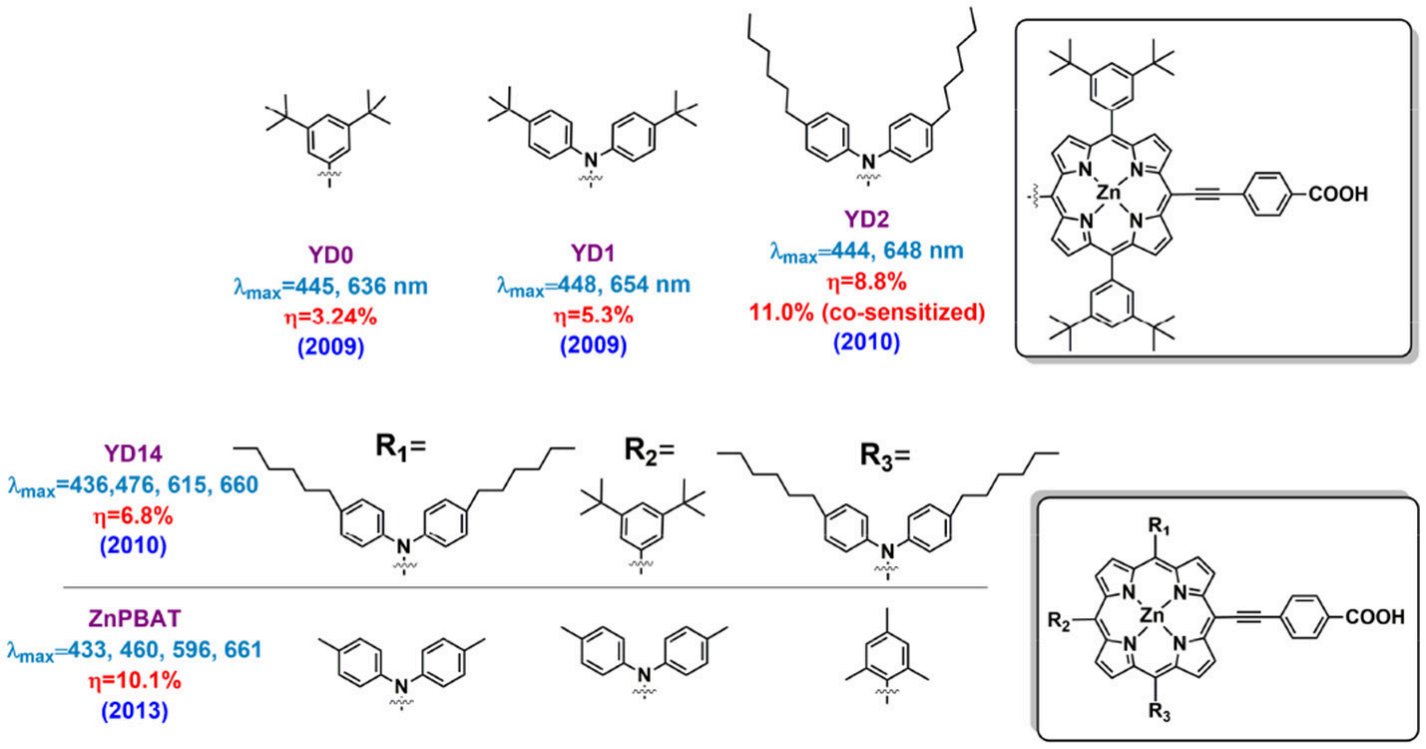
Structure of porphyrins bearing with diphenylamine as donor group.

Featuring with the advantages such as efficiently broadening the absorption area of the porphyrin, fast regeneration reaction with the reductant in redox mediator and so on, diarylamino group is one of the most popular electron-donating group in building D-A porphyrin. To clarify the function of the diarylamino in porphyrin sensitizer, we can take a close look at YD0 and YD1 porphyrins, which have similar molecular structure except that YD1 contains a DPA donor group while YD does not (Figure 5) (Lu et al., 2009). This simple difference led to an obvious variation in the absorption spectra and DSSC performance, being PCE of 2.4% for the YD0 and 6.5% for the YD1. Later impedance spectroscopy (IS) study demonstrated the diarylamino group plays a key role in retarding the I${}_{3}^{-}$ ions from the TiO_2_ surface, which resulted of a significantly reduced charge recombination of YD1 than YD0. Besides, excited-state relaxation dynamics study by femto-second fluorescence decays measurement showed a higher electron injection rate of YD1 sensitized-TiO_2_ than that of YD0. Comparing to YD1, YD2 (Figure 5) contains two more hexyl chains on diphenylamine, which has been proved to be beneficial for dye solubility, suppression molecular aggregation and reducing the recombination reaction between I${}_{3}^{-}$ and TiO_2_. The performance of YD2 devices was improved to 8.8% by Grätzel in the same year as it created, and a higher PCE of 11.0% was achieved by using an organic co-sensitizer to supplement the porphyrin's absorption gap between the B and Q band (around 500 nm) (Bessho et al., 2010). This result is quite impressive because it was the first report on ruthenium-free sensitizer based DSSC achieving efficiency over 11%.

Compared to the ruthenium dye, the narrow B and Q band of typical porphyrins limits the light harvesting properties in DSSC. Especially considered the absorption gap between B and Q bands locates the most intensive region of the solar spectrum (around 500–550 nm), this weakness limits the current density of porphyrin sensitized solar cells. Fortunately, as Imahori et al. pointed out in 2009, elongation of the π conjugation and break the molecular symmetry can broaden and red shift the absorption bands of porphyrins (Imahori et al., 2009). However, this strategy is not always efficient if merely increase the conjugation area or decrease the symmetry of the molecule. Several related reports have shown disappoint results, in which damaged ICT properties could be one of the major reasons. For example, Yeh and Diau designed the YD14, YD15, and YD16 porphyrins (Figure 5) in 2010 (Wu et al., 2010). The YD14 porphyrin has a feature of one more triphenylamine attached onto the 20-position (position accords to Figure 2) than YD15. For YD16 porphyrin, two triphenylamine groups are located at the 5 and 15 *trans*-position and an additional diphenylamine is attached at the 20-position. For the sake of this design, the YD14 shows a split B band at 436 and 476 nm, and the maximum Q band at 660 nm, while they are 440, 488, and 707 nm for the YD15, 451, 600, and 660 nm for the YD16. However, the DSSCs based on these highly asymmetrical and panchromatic porphyrins only exhibited PCE of 4.2% for YD15 and 5.5% for YD16, along with a nearly 20% J_SC_ drop compared with the YD2 device.

In another case, by locating the two diphenylamines to the 5- and 10- positions (*iso-*position according to Figure 2A), Imahori and co-workers successfully broke the molecular symmetry while remained an efficient ICT properties in ZnPBAT (shown in Figure 5), which successfully obtained a remarkable PCE of 10.1% (Kurotobi et al., 2013). This rational design concept based on highly asymmetric, push-pull substitution suggests new possibilities for further improving porphyrin performance. The ZnPBAT porphyrin shows a red-shifted Q band maximum at 661 nm, leading to a broader IPCE spectra and about 2 mA/cm^2^ higher current density at similar condition. It is interesting to note that all the cells reach their highest efficiencies after several days aging under dark. More importantly, the ZnPBAT devices retained 90% of initial performance assessed under continuous AM 1.5G illumination at 25°C.

#### Dialkylamino as electron-donating group

Porphyrins constructed with dialkylaminophenyl groups always show broader light-harvesting area than the diarylamino ones, even the latter possess larger conjugation area. LD14 porphyrin (Figure S1) features with 4-ethynyl-N,N-dimethylaniline as donor group while the acceptor is similar to YD2 (Chou et al., 2016). This porphyrin showed a wide IPCE onset till to 780 nm because of a Q band maximum at 667 nm, impressive high PCE of 10.2% was obtained. By adjusting the alkyl chains in amino group from methyl to octyl, LD16 porphyrin showed even higher performance than the LD14 at same condition, which was supposed to benefit from a successfully retarded charge recombination accomplished by these optimized alkoxyl chains(Wang C.-L. et al., 2012).

Inserting an anthracene between the aniline and the triple bond is also another choice to elongate the conjugation area but remain an efficient ICT, which is demonstrated to be efficient in broadening the IPCE thus increasing the current density of porphyrin based DSSC. Lin and Diau synthesized LD31 porphyrin (Figure S1) with a Q band maximum at 691 nm (Wang et al., 2014a). The significant red-shifted absorption spectrum of LD31 makes a DSSC capable of light-harvesting ability over 800 nm, which showed a PCE of 10% as well.

#### Benzene derivatives as electron-donating group

As proposed in D-A metal-free organic dyes, the function of donor is pushing the electron to the acceptor group, finally to the semi-conductor. However, it's still delusive how important of the electron-donating ability of this group in porphyrin dyes. In most highly efficient dyes, electron-density of the HOMO is mainly distributed at the donor site as from the DFT calculations. In 2011, a series of phenylethynyl-substituted porphyrin sensitizers bearing with nitro, cyano, methoxy, or dimethylamino phenylethynyl substituent were prepared by Lin and Diau, aiming to examine the electron-donating or -withdrawing effects of dyes on their photovoltaic performance (Lo et al., 2010). Along with an increased electron-donating ability, the HOMO and LUMO levels for these four porphyrins negatively shift with a trend of cyano<nitro<methoxy<dimethylamino. The DFT calculation indicates that electrons are effectively injected from the dye to the TiO_2_ for the Me2N-PE1 (Figure S2) and MeO-PE1 upon excitation. As a result, the DSSC performance of these four porphyrins showed a similar trend of the energy levels, in which Me2N-PE1 porphyrin featured with electron-donating group achieved a PCE of 6.1%. These results have provided us a glimpse of the relationship between the donor structure and the energy levels of the porphyrin molecule, and finally the relation to the DSSC photovoltaic properties.

introduction of an additional π-chromophore is another efficient strategy to achieve excellent light-harvesting ability in the visible and Near-IR regions for porphyrin. In 2011, Lin and Diau investigated the phenylethyne, naphthalenylethyne, anthracenylethyne, phenanthrenylethyne, and pyrenylethyne substituted porphyrins (LD1-LD4) and their application in DSSC (Wang et al., 2011). The pyrenylethyne conjugated porphyrin LD4 (Figure S2) showed much improved photovoltaic performance compared with the other porphyrins, giving an J_SC_ of 19.2 mA/cm^2^ and overall efficiency of 10.1%. LD4 was further modified to LWP4 porphyrin by introducing an ethynylanthracene group between the pyrene and porphyrin. However, the absorption spectrum of LWP4 is more like the LAC3 (with only anthracene) instead of LD4 (with only pyrene), showing split B band absorption instead of red-shifted Q band (Wu et al., 2014). The significant performance difference between LD4 and LWP4 based DSSC suggested that there should be a certain length of the donor group.

#### Carbazole and fluorene as electron-donating group

Carbazole groups possess large similarity to the DPA, but with more rigid configuration (Zhao et al., 2016). Considering the “ring fusion” effect, one can expect a lower HOMO energy level of carbazole conjugated porphyrin compared to the DPA porphyrin (Qi et al., 2015). Xie et al. demonstrated that an ethynylene bridge between carbazole and porphyrin causes a decrease in the energy gaps, resulting in a red shifting of the absorption band (Sun et al., 2014; Wang et al., 2014c; Tang et al., 2015). They demonstrated that attachment of long alkyl chains at the *ortho* positions of the *meso*-substituents in porphyrin is an effective strategy to prevent the charge recombination and improve the V_OC_ and J_SC_ values, as well as the cell efficiencies. Thus, a 10.4% of PCE was achieved by XW4 co-sensitized with an organic dye. Not only with high cell efficiencies, they found that the XW4 based devices show satisfactory photostability under simulated solar light.

Attaching multiple electron-donating groups on diphenylamino also has been investigated. For example, Kim and co-workers reported a series of porphyrin sensitizers employed bulky alkoxy group substituted fluorene derivatives as electron donor moieties (Kang et al., 2012, 2013). Benefit from an efficient retardation of charge recombination, the DSSC based on 2,4-ZnP-CN-COOH displayed a PCE of 8.5% with V_OC_ of 739 mV. In 2014, Zhao et al. reported a series of push-pull porphyrin dyes featuring with different numbers of di-fluorene-amine at the donor moiety (Li et al., 2014a,b,c). With a C-N linkage of bis(4-hexylphenyl)amine to 9,9-dihexyl-9H-fluorene to construct the donor group, the ZZX-N4 exhibited better DSSC performance than the other analogs. They found that extension of π-conjugation area of donor group can hardly achieve a positive effect on the absorption spectra, as well as the IPCE of the DSSC devices.

#### Porphyrin dimer

To generate a large photocurrent response in DSSC, sensitizers should own broad and intense absorption. Porphyrin arrays exhibit strong electronic coupling between porphyrin rings, resulting in splitting of the B band and broadening of the Q band (Campbell et al., 2004). Indeed, the linkage of the porphyrins is a criterion factor determines the array's ICT therefore their spectroscopy properties. For example, in 2010, Yeh and Diau reported the triple bond linked porphyrin (YDD0) exhibited lower performance than single bond linkage porphyrin (YDD1) (4.1 vs. 5.2%), despite of a broader light-harvesting area of former (Mai et al., 2010). Calculation results suggested that the nearly planar structure of YDD0 facilitates π-conjugation between two porphyrin macrocycles, explaining the broader absorption area than YDD1. Whereas, the down-shift LUMO of YDD0 decreases the electron injection driving force. The planar geometry also induces serious dye aggregates, leading to a more non-radiation recombination of the excited dye. These factors eventually decrease the performance of the final device. In 2011, Segawa et al. designed a porphyrin dimer DTBC (DTBC in Figure S3) (Liu et al., 2011, 2012). The IPCE of DTBC device shows an onset to 900 nm and steeply reaches almost a plateau value close to 60% from around 400 nm until 800 nm. By employing high concentration of tBP to down-shift the conduction band of TiO_2_, they succeeded in enhancing the electron-injection efficiency.

The JY07 porphyrin (Figure S3) features a double D-A branched structure, which was expect to increase the paths for efficient electron extraction (Zhang et al., 2015). JY07 with orthogonal conformation was also supposed to minimize the molecular aggregation on TiO_2_ and suppress charge recombination in DSSC device. The V_OC_ of JY07 porphyrin was 650 mV, which was impressive for porphyrin dimer DSSC. However, inefficient conjugation of the triphenylamines with the porphyrin dimers limited the IPCE of JY07 to 725 nm.

Meanwhile, porphyrin dimers were expected to be ideal co-sensitizer to capture the solar energy in the longer wavelength area (Wu et al., 2012; Shiu et al., 2015). In 2012, Diau et al. reported a judiciously co-sensitization engineering of TiO_2_ films with an organic dye, YD2-oC8 and dimer YDD6. The devices showed panchromatic spectral features in the IPCE spectrum up to 850 nm, while the PCE was 10.4%. These porphyrin-based co-sensitization systems showed only 10–15% degradation in 300 h at 25–30°C. They also reported that the porphyrins intend to desorption from the nanocrystalline particles when device temperature got higher than 60°C. Therefore, modification on the anchor group between the porphyrin and nanocrystalline become vital for improving the thermal stability of porphyrin sensitizers. We will discuss this part in the next section.

#### Other donor groups

In the early years, the research of electron-donating groups in porphyrin dyes was major focused on arylamino or the derivatives. It's indeed useful in designing useful porphyrins for DSSC, such as YD2 (bis(4-hexylphenyl)amine), YD2-o-C8 (bis(4-hexylphenyl)amine), ZnPBAT (di-*p*-tolylamine), GY50 (bis(4-hexylphenyl)amine) (Yella et al., 2014), SM315 (bulky bis(2′,4′-bis(hexyloxy)-[1′-biphenyl]-4-yl)amine donor) *etc*. Recently, researchers started to put some eggs to the other bucket (Yang et al., 2014; Qi et al., 2018). For example, some electron-donating groups in organic sensitizer were successfully applied in porphyrin, such as N-annulated perylene (**26** in Figure 4) (Jiao et al., 2011; Luo et al., 2013, 2016), 2-diphenylaminothiophene (**23** in Figure 4) (Wang et al., 2014d), indoline (**24** in Figure 4) (Pellej et al., 2014), phenothiazine (**31** in Figure 4) (Xie et al., 2015), and even ferrocene (van der Salm et al., 2015). Some concepts from low-band-gap polymer were successfully applied in adjusting the absorption area and energy alignment of the porphyrins, such as weak donor-strong acceptor (Zhou et al., 2012; Bai et al., 2018; Lin et al., 2018; Xie et al., 2018), which will be discussed in the following part.

### The acceptor groups

**Figure 6:**
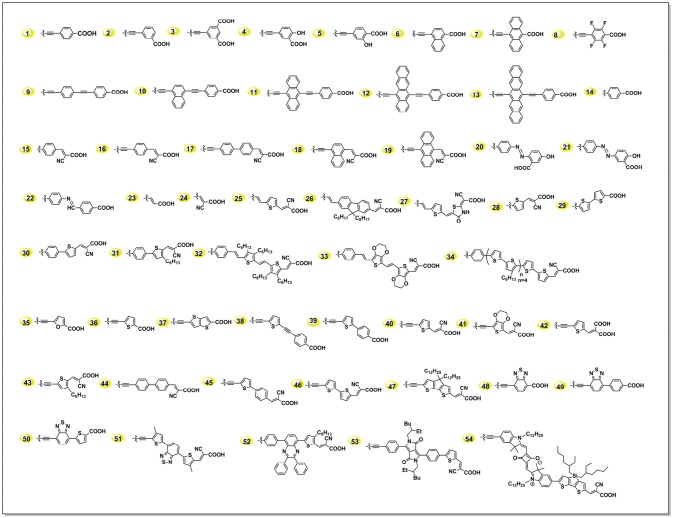
A summary of some acceptor groups in D-A porphyrin sensitizers.

The acceptor structure in a push-pull porphyrin determines not only the charge injection, separation and transfer processes, but also the adsorption geometry of dye on TiO_2_ (Imahori et al., 2010; Ye et al., 2013; Hayashi et al., 2015). As we summarized in Figure 6, an acceptor group usually constructs with a triple bond, which is used to connect to the porphyrin; an aromatic linker group, which is to transfer the electron to the anchor; and carboxyl acid, which grafts to the semi-conductor. For example, in acceptor **1** in Figure 6, we can regard the phenyl unit as linker. In this article, we discuss the linker group first, and then give a summary of the anchoring group.

The primary principle for designing efficient dyes is building the framework of push-pull structure within the molecule, which intrinsically benefits to the ICT process thus charge separation. Porphyrin is a kind of M-N4 macrocycles, whereas this metal-coordinated molecule works more like organic molecules. As pioneered by Diau and Yeh in 2009, 4-ethynylbenzoic acid (**1** in Figure 6) was demonstrated to be one of the most successful and popular acceptors. Series of highly efficient porphyrins sensitizers were constructed with this rigid acceptor, including the benchmark porphyrins, i.e., LD14, YD2-o-C8. Since this unique acceptor was created several years ago and has already been deeply investigated, we will majorly focus on the alternatives in the later discussion.

#### Secondary chromophores conjugated acceptor

Insertion of secondary chromophores into the donor framework was reported to be efficient in broadening the IPCE while maintain the V_OC_. Following this idea, Lin and Diau systematically tuned the absorption spectra by inserting acenes into the acceptor (Figure 6) (Lin et al., 2009). The best performance was achieved with LAC-3 porphyrin (5.4%), which bears with an anthracene as part of the linker. And the photovoltaic performance was decreased when it was tetracene or pentacene, which can be attributed to the inappropriate LUMO levels.

#### Electron-withdrawing group in acceptor

Ideally, enhance the donor's electron-donating ability and/or the acceptor's electron-withdrawing ability is beneficial to the ICT of the sensitizer, thus increases the injection efficiency and reduce the charge recombination (Wang et al., 2018). Yeh and Diau used the acrylonitrile group to build the acceptor (Lee et al., 2009). However, this porphyrin presented serious charge recombination and low injection efficiency. The authors suggested that the superior electron- withdrawing ability of cyano group first pull the electrons and then transfer them back into the porphyrin core. Another case is the ZnPF and ZnPH porphyrins (shown in Figure S4), which were reported by Imahori et al. in 2011 (Mathew et al., 2011; Sakurada et al., 2014). ZnPF featured with 4 fluorine atoms substituted on the phenyl linker, supposed to enhance the electron-withdrawing ability of the acceptor. Unfortunately, the ZnPF based DSSC showed lower performance than ZnPH, which was attributed to the more serious recombination reaction caused by the fluorine atoms.

#### Thiophene and derivatives conjugated acceptor

**Figure 7:**
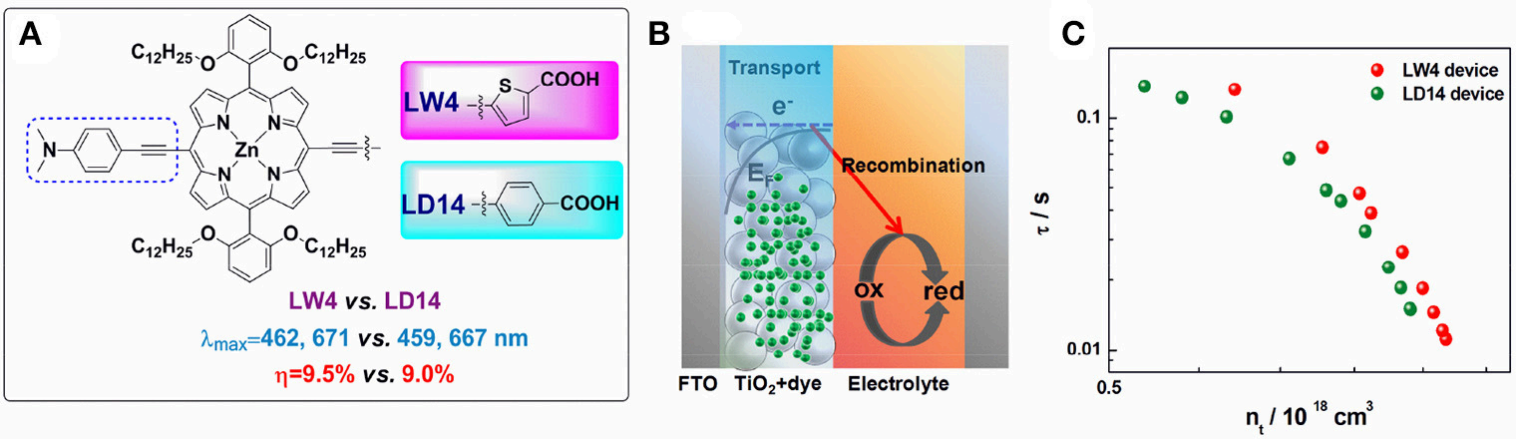
**(A)** Molecular structure of LW4 and LD14 porphyrins; **(B)** schematic of electron transfer and recombination processes in DSSC; **(C)** the relationship of recombination lifetime and photo-induced charge density for LD14 and LW4 devices.

The linker between the porphyrin and anchoring group plays a key role in modulating D-A electronic coupling as well as the magnitude of the change in dipole moment (Liu et al., 2009; Arja et al., 2018). Thiophene and its derivatives have been widely studied as linkers in organic dyes or low band-gap polymer (Zhou et al., 2015). In 2013, an efficient LW4 porphyrin employed 5-ethynylthiophene-2-carboxylic acid as acceptor was reported by Wang et al. (Lu et al., 2013a). LW4 shows similar spectral properties in comparison with its analog LD14, while the solar cells showed superior a performance (9.5 vs. 9.0%) at similar conditions. Transient photovoltage decay and charge extraction measurements revealed a prolonged recombination lifetime of LW4 devices (shown in Figure 7), indicating a less interfacial charge recombination process in the devices.

Within a rigid structure between porphyrin and carboxyl acid, sensitizers LW17, LW18, and LW19 (Figure S5) successfully indicated information on how the hetero-aromatic linker affect the DSSC performance, in terms of conjugation length, adsorption angle on TiO_2_ (Lu et al., 2014a). They all have similar donor, porphyrin core and anchoring group structure, while only differentiate the linker group with phenyl, thiophene, and elongated 2-phenylthiophene. The absorption spectra (Q band maximum presented a stepwise red-shift as 674, 678, 683 nm, but obviously differentiate in DSSC performance. The resulted LW18 devices showed 8.7% PCE. Comparing to the LW17 or LW19 devices, LW18 devices presented longer electron recombination lifetime in LW18 device as revealed by transient decay measurements. This result also demonstrates that the recombination reaction on the TiO_2_ interface is highly related to the linker groups.

**Figure 8:**
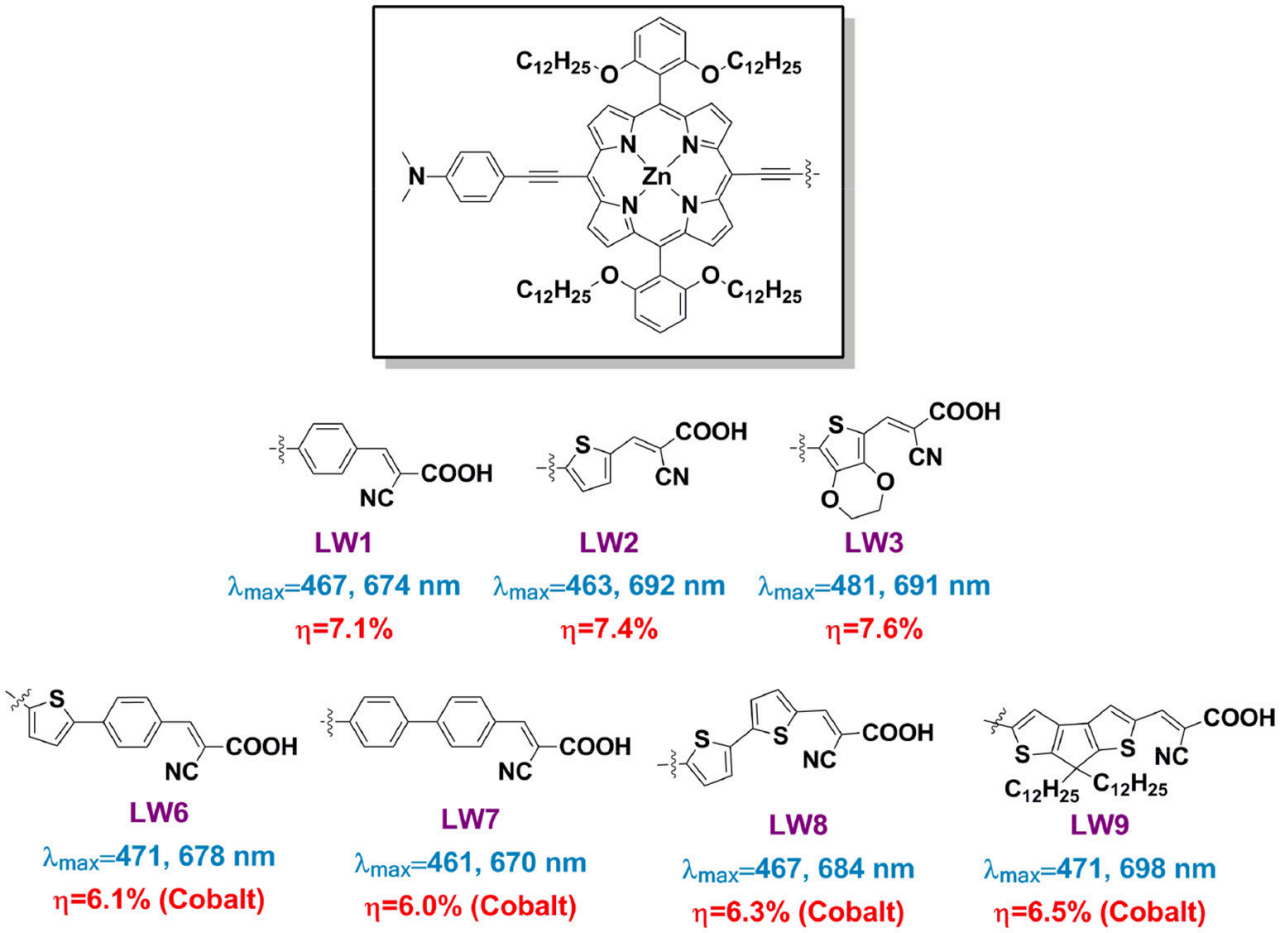
Structure of some porphyrins featured with variety linker.

One of the attractions in DSSC research is the controllable sensitizers' molecular structure through synthesis approach. When investigate how the linkers influence a porphyrin's spectral and photovoltaic properties, one can make the porphyrins bearing with exactly a same donor, porphyrin ring, and anchor, but only varied the linker group, such as benzene (LW1), thiophene (LW2), 3,4-ethylenedioxythiophene (EDOT, LW3), 2-phenylthiophene (LW6), 1,1′-biphenyl (LW7), 2,2′-bithiophene (LW8), and cyclopenta-[1,2-b:5,4-b′]dithiophene (CPDT, LW9) (Figure 8) (Lu et al., 2013a, 2014b,c). The Q band maximum of LW2 porphyrin can be red-shifted to 692 nm by 18 nm in comparison with LW1, whereas the LW6 porphyrin blue-shifts 14 nm compared with LW2 despite extended conjugation length. These results strongly suggested that the light-harvesting ability of porphyrin is not simply determined by the molecule's conjugation area, but also other factors, such as dipole moments, ICT process. Devices based on porphyrin LW2 and LW3 presented PCE of 7.4% and 7.6%, higher than that of LW1 (7.1%) but lower than LD14 (9.0%). Impedance spectra and transient decay measurements suggested this difference was ascribed to the variation of recombination reaction at the TiO_2_/porphyrin/electrolyte interface. Bisquert et al. suggested that the floppy structure of cyanoacetic acid of the anchoring group tilted the molecules on TiO_2_ film, which increased the aggregation of the molecules thus decreased its IPCE values and V_OC_ (Ripolles-Sanchis et al., 2012).

The linker's electron negativity was also reported to capable of influencing the porphyrin's properties. For example, when the linker switched from 1,1′-biphenyl to 2,2′-bithiophene and cyclopenta-[1,2-b:5,4-b′]dithiophene (CPDT), the absorption area and DSSC device performance for LW7, LW8, LW9 broadens and increases stepwise (Figure S6). The LUMO of these porphyrins down shift when the linker changed from bi-phenyl to CPDT. Considering an increase electron-donating ability of these linkers, it was supposed that a D-π-D-A structure might be benefit to red-shift and broaden the porphyrin sensitizer and improve the device performance (Lu et al., 2014b).

After the first report by Wang *etc*., a variety of thiophene conjugated rigid acceptors have been created (Sreenivasu et al., 2014; Zhang M.-D. et al., 2014). Interesting to note that furan and bithiophene linked acceptor functionated porphyrins exhibit broader absorption area than that of thiophene but dramatically dropped performance (2.8 and 1.4%). In 2015, Zhao reported ZZX-N8 porphyrin (Figure S7) utilized thieno[3,2-b]thiophene to substituted the thiophene (ZZX-N7) to expand the conjugation area in the linker place, the conjugated porphyrin achieved a slight higher PCE than the porphyrin conjugated with thiophene (7.8 vs. 7.5%) (Li et al., 2015). Choi et al. reported a porphyrin (ZnP-2 in Figure S7) featured with 4-((5-ethynylthiophen-2-yl)ethynyl)benzoic acid acceptor, in which a triple bond was inserted between the thiophene and phenyl (Chae et al., 2014). These two triple bonds between DPA and carboxylic acid group was reported to suppress the interface charge recombination reaction, delivering a 6.7% PCE of DSSC.

#### Electron-deficient unit conjugated acceptor

**Figure 9:**
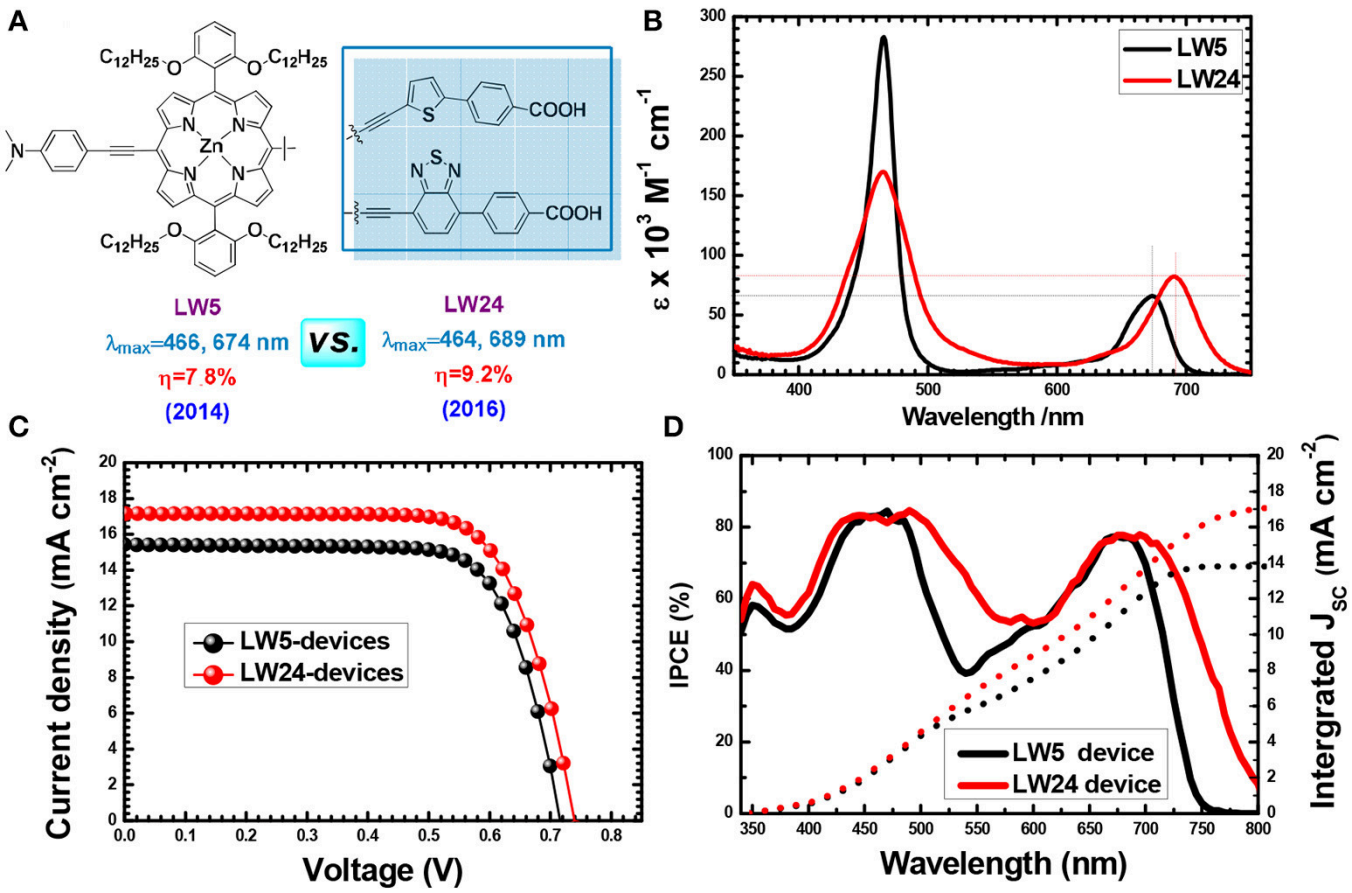
Porphyrin conjugated with electron-rich or electron-deficient unit in the acceptor **(A)** Molecular structure, **(B)** absorption spectra of, **(C)** J-V curves and, **(D)** IPCE of LW5 and LW24 devices.

The strategy of introducing electron-deficient units as the auxiliary electron acceptor for push-pull porphyrins was the most successful progress in recent years. Benzothiadiazole (BTD) (Zhou et al., 2012; Xu et al., 2014; Lu F. et al., 2017), 2,3-diphenylquinoxaline (DPQ) (Fan et al., 2015), diketopyrrolopyrrole (DPP) (Warnan et al., 2012), and squaraine (SQ) (Jradi et al., 2015) *etc*. have been extensively exploited to re-construct D-A porphyrin. Studies demonstrated that integration of those proquinoidal units into the porphyrin structure causes strong perturbations to the electronic structure of the porphyrin ring, which lead to a broadening and red shifting of porphyrin's both B and Q-bands absorbance. For example, we synthesized analog porphyrins LW5 and LW24 (Figure 9) to analysis the benefit of BTD unit between the macrocycle and benzoic acid (Lu et al., 2015a, 2016). The LW5 and LW24 dyes feature with similar structure, except that the linker is an electron-rich unit thiophene for the former, whilst it is electron-deficient BTD unit for the latter. The LW24 porphyrin exhibits nearly 50 nm absorption onset red-shifts from 750 to 800 nm, along with more than 30% Q band absorption intensity compared with LW5. Finally, the LW24 devices presented 9.21% PCE, while it was 8.16% for the LW5 porphyrin.

**Figure 10:**
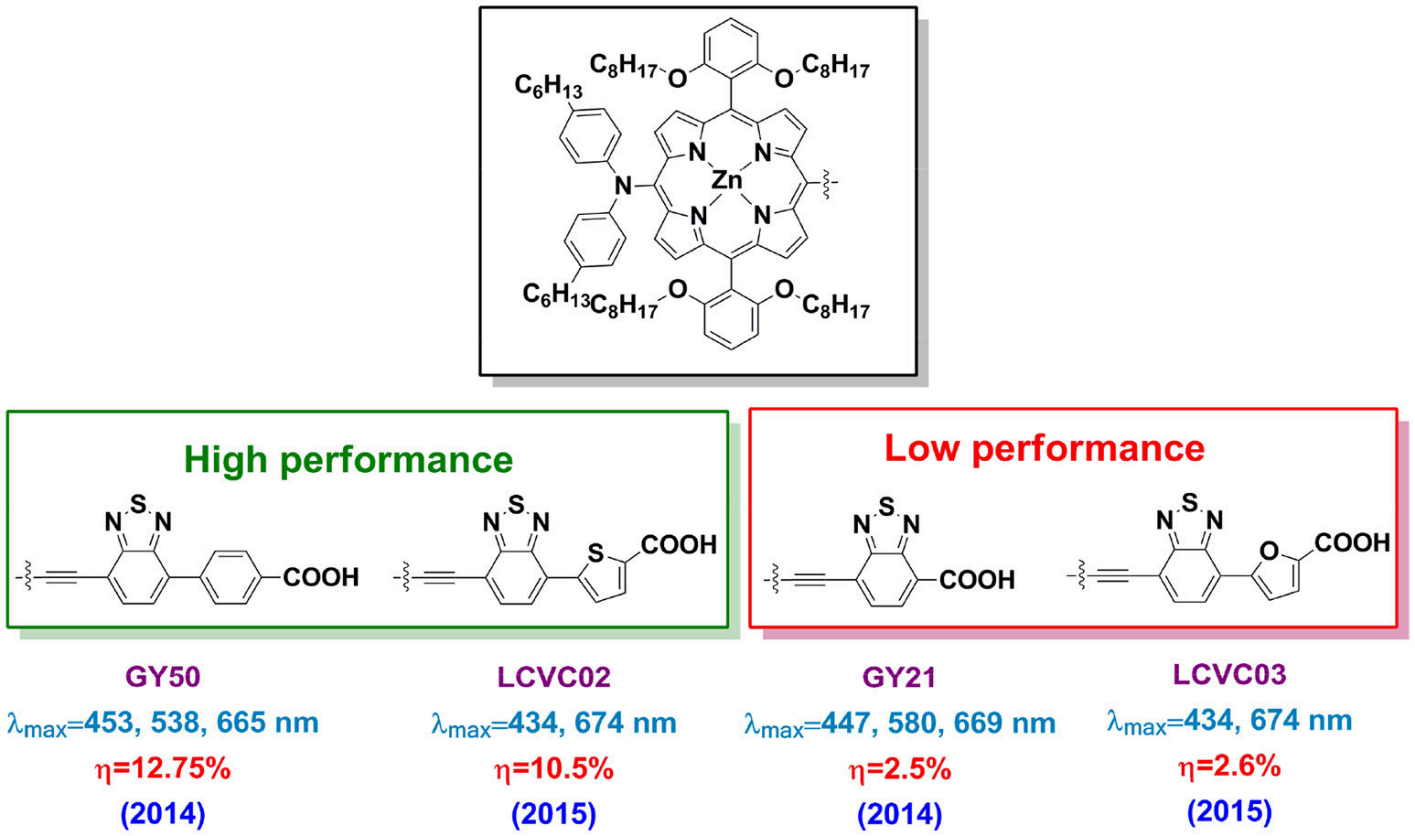
Structure of some porphyrins featured with electron-deficient unit.

It is worth to note that the BTD-conjugated porphyrins not always work efficient for DSSC application. For example, if the BTD linked between the porphyrin ring and benzoic acid, the obtained GY50 porphyrin based DSSC reached 12.8% PCE (Figure 10). Whereas it is 2.5% for the analog porphyrin GY21 (BTD connects the porphyrin and carboxyl acid directly) based DSSC despite of a similar absorption property (Yella et al., 2014). This performance difference suggests that locate the electron-deficient directly to the anchor is not a good idea. It is also criteria to choose the aromatic unit between the BTD and anchor. Take the LCVC02 and LCVC03 porphyrins (Figure 10) as examples, only a single atom (O to S) in the linker change switches-on the efficiency from 3% to over 10% (Cabau et al., 2015). Wang and co-workers investigated the suitable position for an auxiliary electron-deficient unit in the porphyrin sensitizers (Fan et al., 2015). A 50% improved PCE was reported when places the DPQ in the acceptor instead of the donor part (6.0 vs. 4.0%). In Table 1, we summarized the absorption peaks and DSSC performance of some porphyrins featured with electron-deficient linker.

**Table 1:** The B and Q band absorption peaks of porphyrin sensitizers featured with electron-deficient linker, and DSSC performance (shown without co-sensitizer, “*” is for the cobalt-based DSSC performance).

**Dye**	**B and Q band peaks (nm)**	**PCE (%)**	**Year**	**References**
GY21	447, 558, 669	2.5	2014	Yella et al., 2014
GY50	453, 538, 665	12.8^*^	2014	Yella et al., 2014
SM315	440, 454, 581; 668	13.0^*^	2014	Mathew et al., 2014
LCVC02	434; 674	10.5	2015	Cabau et al., 2015
LCVC03	434; 674	2.6	2015	Cabau et al., 2015
FNE58	425, 557; 597	6.0	2015	Fan et al., 2015
XW17	469, 650, 693	9.5	2015	Tang et al., 2015
XW11	465, 622; 683	9.3	2015	Xie et al., 2015
LWP14	477, 696	10.3^*^	2015	Ali et al., 2016
LW24	464, 689	9.21	2016	Lu et al., 2016
WW9	435, 474, 686	9.2	2016	Luo et al., 2016

#### Acceptors without alkynyl unit

By introducing benzoic acid or corresponded ester before constructing porphine macrocyle, porphyrin dyes without triple bond in the acceptor can be obtained (acceptor **18** in Figure 6) (Hayashi et al., 2015). Kim and co-workers reported series of push-pull porphyrins with benzoic acid or 2-cyano-3-phenylacrylic acid (acceptor **19** in Figure 6) (Seo et al., 2012). HKK-Por1 incorporated with 4-benzoic acid obtained a PCE of 5.9%, while 2,4-ZnP-CNCOOH porphyrin with 2-cyano-3-phenylacrylic acid acceptor achieved remarkable 8.5% with the HC-A1 co-adsorbent (Kang et al., 2013).

Through one-pot with two-step condensation reactions, followed by high-yielding metalation and hydrolysis steps, Hung and Diau synthesized a series of A2B2 porphyrin, in which 2 acceptors were used to enhance the dye-loading amount and control the adsorption geometry as well (Ambre et al., 2012, 2013; Luo et al., 2015). They placed the donors and acceptors at macrcycle's *cis*-position. ATR-FTIR spectroscopy confirmed that those A2B2 porphyrins chelated to the TiO_2_ surface with a double-arm-binding mode. The highest efficiency was below 5% but still provided a solution to enhance dye attachment and photo-stability.

### Anchoring group of push-pull porphyrin

In most porphyrin sensitizers, as well as pure-organic or ruthenium sensitizers, carboxylic acid groups have been widely used as anchoring groups to graft the dyes onto the TiO_2_ surface. Not only that carboxylic acid groups can be easily introduced into molecules through a variety of readily accessible precursors, they also offer very good electronic communication between the dye and TiO_2_ by forming a strong ester linkage with the Brønsted acid sites (surface bound hydroxyl groups) of the TiO_2_ surface. Nevertheless, there is an ongoing quest for alternative anchoring groups on account of several reasons. For example, it was reported the H^+^ can intercalate into the TiO_2_ surface during the dye-dipping process, which was reported to induce a positive shift (vs. *NHE*) of the TiO_2_ conduction band edge and decrease the V_OC_ of the devices (Ooyama et al., 2011, 2016). Meanwhile, porphyrins with carboxyl acid have a tendency to dissociate slowly from the TiO_2_ surface under long time illumination or at high temperature, losing their capability to convert photons to electrons, and finally reduces the long-term stability of DSSC (Zhang et al., 2017). Thus, pyridine (Lu et al., 2013b; Sharma et al., 2013), 8-hydroxylquinoline (He et al., 2012b), perylene (Jiao et al., 2011), naphthalene (Lee et al., 2011), and other units (Mai et al., 2015) were explored as alternatives of carboxylic acid (Figure S8).

**Figure 11:**
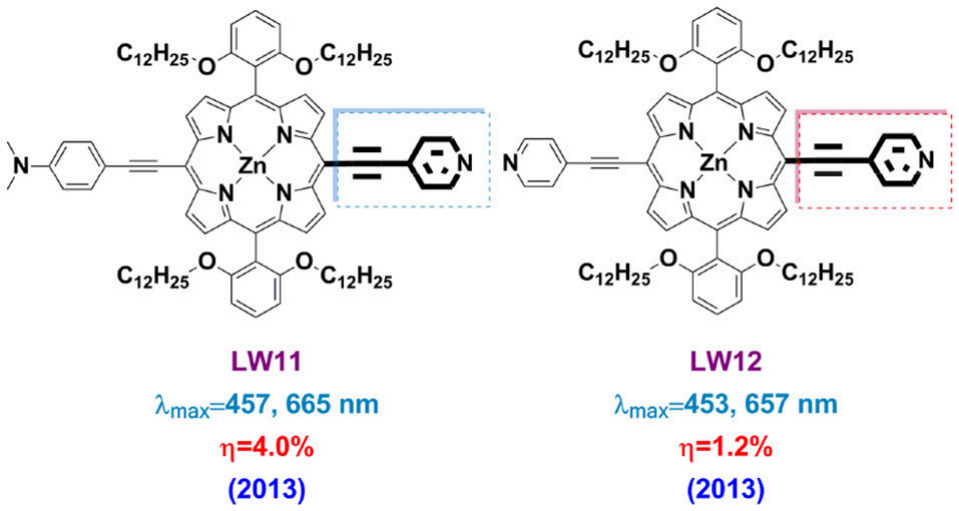
Porphyrin sensitizers with pyridine as anchoring group.

Pyridine can coordinate with the Lewis acid sites of the TiO_2_ surface, providing good electron communication in-between and lead to very efficient electron injection. Sharma and co-workers reported P-1, P-2, and P-3 porphyrin, bearing with one, two and four pyridyl groups, respectively, in the meso positions, acting as anchoring groups. PCE of 3.90% was achieved based on P-2 porphyrin (with 2 pyridine and 2 carboxymethylphenyl at the *meso*-positions) and boosted to 6.1% after the photoanode was treated with formic acid (Sharma et al., 2013). Wang et al. demonstrated the pyridine can construct an efficient push-pull porphyrin by synthesizing LW11 and LW12 (Figure 11) in 2013 (Lu et al., 2013b). LW11 porphyrin featured with asymmetry character, in which 4-ethynyl- N,N-dimethylaniline was donor whilst 4-ethynylpyridine as acceptor. For LW12 porphyrin, two 4-ethynylpyridines were attached at the *trans*-position of the macrocycle. The spectral properties of LW11 and LD14 are similar, whereas the LW12 presents obvious blue-shifted absorption and emission spectra. These results suggested that 4-ethynylpyridine successfully played the acceptor role as a D-A sensitizer. The LW11 porphyrin-based device achieved a 4.0% PCE, which is much higher than the LW12 devices (1.2%). Dye-loading measurements told that the dye amount of LD14 devices is nearly 5-folds of LW11 device, which should be a major reason for the lower DSSC performance of the latter.

He *et al* introduced the 8-hydroxylquinoline as anchoring group for porphyrins, in which N and O atoms were used to chelate to metal ions forming stable complexes (He et al., 2012a). They further tested the adsorption ability by immersion the sensitized TiO_2_ film in acetic acid solution in acetonitrile, in which TPPZn-OQ porphyrin showed much superior stability than its carboxyl acid analog (Si et al., 2014). Yeh and Grätzel reported porphyrins MH2 and MH3, in which hydroxyl or carboxyl acid was attached to the 2-position of the pyridine to enhance the adsorption ability (Mai et al., 2015). It is worth noting that MH2 porphyrin showed a superior long-term stability in comparison with the carboxyl acid functionalized YD2-o-C8. Using an ionic-liquid based electrolyte, after a period of 1,000 h under simulated AM 1.5 G (100 mW cm^−2^) at 60°C, MH2 maintained around 90% performance, whereas it was 85% for YD2-o-C8 devices. This result suggested that 2-carboxypyridyl outperforms the 4-carboxyphenyl as the anchoring group and acceptor.

### Modification on the porphyrin macrocycle

Normally, porphyrin based DSSCs show comparable or even higher J_SC_ with that of ruthenium complex, but always lower V_OC_, which is the major bottleneck for further improvement in device performance. This issue can be ascribed to two major reasons: significant aggregation for porphyrin sensitizers and the recombination reaction between reductant in the electrolyte and the positively charged metal-center (usually zinc) of the porphyrin core. Both reasons diminish the electron lifetime in the devices and increase the charge recombination, which result of decreased V_OC_.

**Figure 12:**
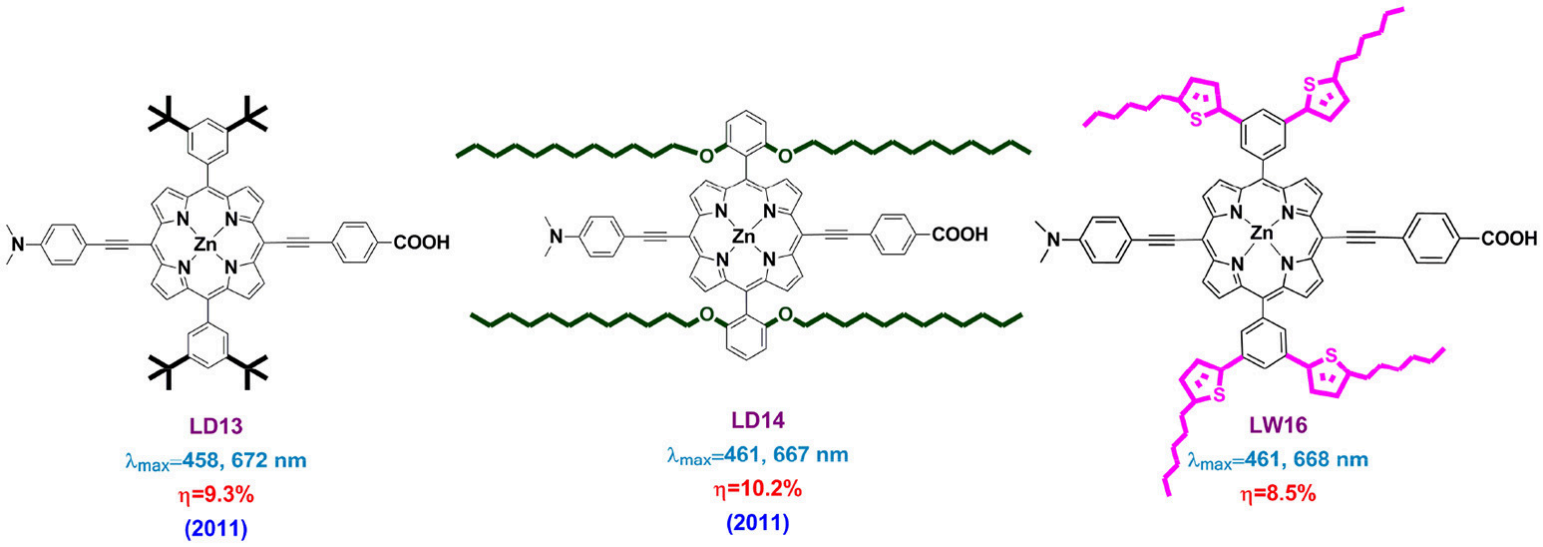
Molecular structure of LD13, LD14, and LW16 porphyrins.

In 2011, Lin and Diau introduced long alkoxyl chains to protect the porphyrin core from charge recombination and also to decrease the dye aggregation (LD13 and LD14 in Figure 12) (Chang et al., 2011). The newly designed LD14 porphyrin featured with two phenyl substituents bearing two dodecoxyl (-OC_12_H_25_) chains at the *ortho* position of each phenyl group. The devices based on LD14 showed an impressive 10.2% PCE with a V_OC_ of 736 mV, while it was 9.3% and 697 mV for LD13 devices. Deeply analysis suggested that this improvement was majorly ascribed to the up-shifted *E*_*CB*_ of TiO_2_ upon dye adsorption, as well as the reduced charge recombination at the electrolyte/TiO_2_ interface. The authors supposed that the four dodecoxyl chains in the devices play a key role in wrapping the porphyrin core in a shape that prevents dye aggregation effectively and forms a blocking layer on the TiO_2_ surface.

Indeed, taking the advantage of long alkoxyl chain in retarding the recombination reaction in the devices, Grätzel, Yeh, Diau and co-workers modified the benchmark porphyrin YD2 to YD2-o-C8. The latter porphyrin created the PCE record of 12.3% at 2011, a breakthrough waited for many years in DSSC community (Yella et al., 2011). It is demonstrated that the advantages of YD2-o-C8 based devices is their significantly prolonged electron lifetime compared with that of YD2, not only in cobalt complex but also iodine-based electrolyte.

One may expect this porphyrin core “envelope” strategy to combine both the porphyrin core protection and conjugation area extension, thus improve both the V_OC_ and J_SC_. For example, LW16 porphyrin, rational designed with four 2-hexthiophenes at the *meta*-positions of each *meso*-phenyl ring attained PCE of 8.5%, while it was 6.9 and 8.2% for the analogs LW15 (four octyloxy) and LW14 (none alkoxyl) (Lu et al., 2015b). However, LW14, LW15, and LW16 porphyrins showed very similar light-harvesting ability, indicating a less effect of conjugation expansion on the *meso*-phenyl.

In quest of low-cost sensitizers, He et al. reported a two-step synthesized zinc porphyrin (DMPZn-C2-COOH) with an acrylic acid at the *meso* position, which achieved 5.1% efficiency (He et al., 2012b). Yeh et al. recently reported the Y1A1 porphyrin, which was large-scalable by simply synthesis without using n-butyllithium (Wang et al., 2014b). A power conversion efficiency of 9.2% was achieved.

## Conclusion and outlook

In order to solve the increasing energy demanding and environmental pollution problems, development of photovoltaic devices for solar power conversion is of great significance. Recently, the metal halide perovskite absorbers in the form of thin film was reported to be able to fulfil complete photo absorption in the visible and NIR region, which have been a hot spot in solar cell research field (Huang et al., 2017; Lu J. et al., 2017). However, the perovskite solar cells are sensitive to the environment stimuli (including oxygen, moisture, UV-exposure, temperature) due to their low chemical instability. In the meantime, DSSC could offer a reliable candidate which deserves much research attention. The progress of DSSC photovoltaic performance over the past 6 years has evidenced focused on a rational design of D-A structured porphyrin dyes, as can be evidenced from Table 2. Take the recent DSSC PCE record improvement from 12.3 to 13.0% as an example, the major contribution came from optimizing the YD2-o-C8 porphyrin structure on both donor and acceptor positions.

**Table 2:** Best performing porphyrins in DSSC achieved a PCE over 9% under simulated AM1.5G sunlight.

**Entry**	**Dye**	**PCE (%)**	**References**
1	SM315	13^*^	Yella et al., 2011
2	GY50	12.75^*^	Yella et al., 2014
3	YD2-OC8+Y123	12.3^*^	Yella et al., 2011
4	SM371	12^*^	Mathew et al., 2014
5	XW11+WS-5	11.5	Xie et al., 2015
6	YD2+D205	11	Bessho et al., 2010
7	XW17 + WS-5	10.9	Tang et al., 2015
8	XS3+XW4	10.75	Sun et al., 2014
9	WW6	10.5^*^	Luo et al., 2013
10	XW4+C1	10.45	Wang et al., 2014c
11	YD2-OC8+CD4+YDD6	10.4	Wu et al., 2012
12	LD14+LDD1	10.4	Shiu et al., 2015
13	LCVC02	10.4	Cabau et al., 2015
14	LWP14	10.3^*^	Wang et al., 2016
15	LD31+AN4	10.26	Wang et al., 2014a
16	LD16	10.24	Wang C.-L. et al., 2012
17	LD14	10.17	Chang et al., 2011
18	ZnPBAT	10.1	Kurotobi et al., 2013
19	LD4	10.06	Wang et al., 2011
20	LWP1	9.73	Wu et al., 2014
21	LD14+H2LD14+AN-3	9.72	Wang et al., 2014b
22	LW4	9.53	Favereau et al., 2013
23	Y1A1	9.22	Guo et al., 2016
24	LW24	9.21	Lu et al., 2016
25	LD12+CD5	9.0	Lan et al., 2012

(* *denotes the use of cobalt-complex electrolyte)*.

**Figure 13:**
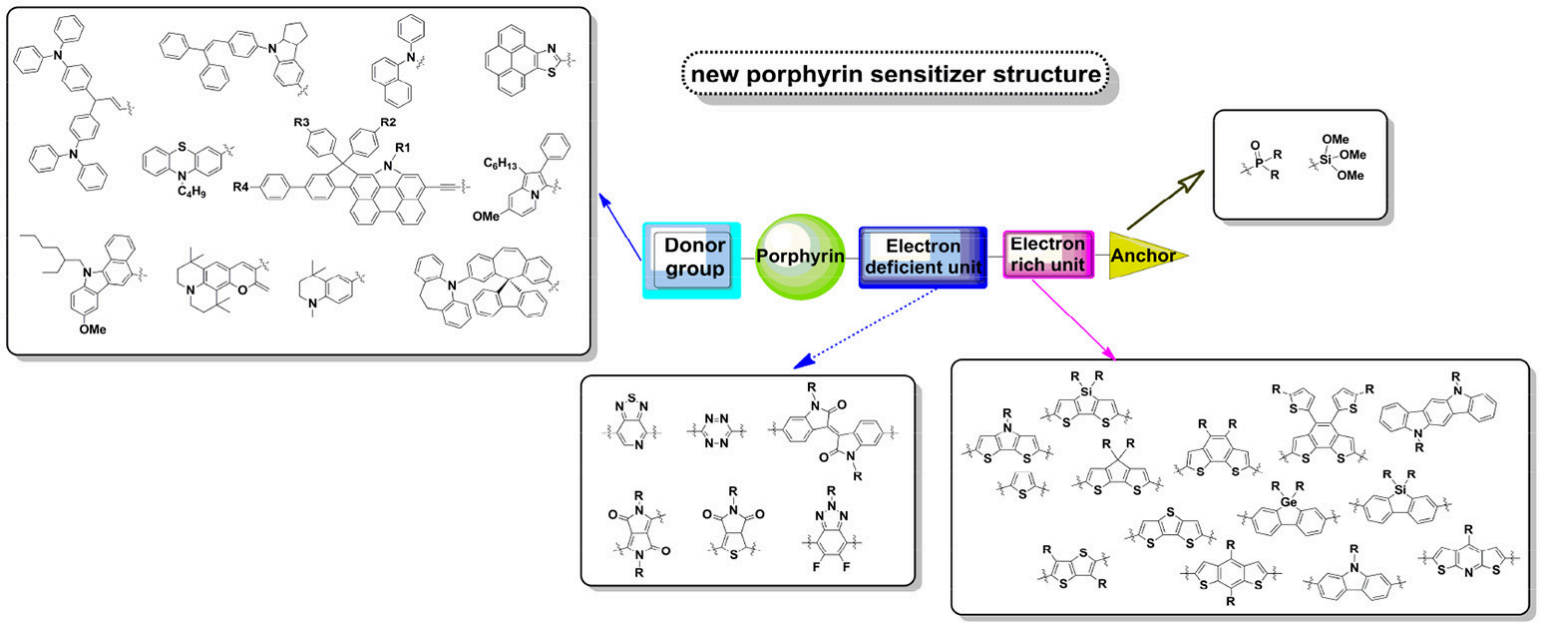
Design of new porphyrin sensitizers.

Deeply looking into the SM315 or other highly efficient derivatives, we may envisage the linker to two parts. One is the electron-deficient unit BTD connecting to the macrocycle, while the other one is electron-rich unit connecting the electron-deficient unit and anchor. Indeed, in the other field of donor-acceptor polymer, it has been demonstrated that a smaller band gap and low-lying HOMO energy level can be obtained with a “weak donor-strong acceptor” strategy, whereas the charge mobility, molecular interaction, and stability can also be adjusted by varying the electron donating ability of the donor moiety and the electron affinity of the acceptor moiety. In this case, various electron-donating units and electron-accepting units (as we summarized some of these groups in Figure 13), ranging from weak electron-donating (or withdrawing) ability to strong have been designed and synthesized to improve the performance of polymer solar cell. Some common-used units in low-band gap polymer have already been adopted into pure-organic or porphyrin sensitizers, which present excellent spectral and photovoltaic properties (such as CPDT, BTD, DPP *etc*). Therefore, we propose that there should be a further improvement of DSSC performance after we make a deep combination of porphyrin with low band-gap polymers.

The anchoring group also plays a vital role in porphyrin sensitizer when we consider the desorption issue of porphyrin under working temperature. Phosphonic, 2-(1,1-dicyano-methylene)rhodanine, silyl-anchor and so on were proved to own strong anchoring ability on semi-conductor, which could be promising candidates for the future stable porphyrin dyes.

As discussed above, the meso-donor group is likely electronically isolated with the porphyrin macrocycle. Thus, instead of expecting broader light harvesting area by tuning the donor structure, future work could be more focused on developing new donor groups that could react fast with the redox couple and retard the recombination. We suppose the many donor parts from highly efficient organic sensitizers are promising for designing new porphyrin sensitizer (we summarized some of these groups in Figure 13). As a success example, the XW11 porphyrin reported by Xie et al., in which phenothiazine derivatives was known as popular donor group to construct metal-free organic dye. The XW11 created new iodine based DSSC PCE of 11.5%. N-annulated perylene (NP) conjugated porphyrin attained over 10% PCE. The VC-70 porphyrin incorporated with indoline donor group also exhibited impressive DSSC performance.

Inspired by the pivotal role chlorophylls in energy and electron transfer processes in the active site of photosynthetic plants and bacteria, porphyrins are recently considered as potential and promising candidates for highly efficient DSSC. In this review, we have made an overall discussion of the porphyrin sensitizers from the molecular structure point of view. To make a further improvement for porphyrin based DSSC, we propose following issues should be considered when design the new porphyrin structures:

1. The light harvesting area of most porphyrin sensitizers is not broad enough to cover the solar spectrum. The DSSC device performance in a cobalt-electrolyte system can be estimated to be 19% if the total energy loss in potential can be controlled to <500 mV while the IPCE onset is extended to 920 nm. However, the champion dye SM315 owns a Q band maximum of 668 nm and IPCE onset of 780 nm. Fortunately, extending the IPCE of porphyrin based DSSC could be realized by tailoring the donor-acceptor structure of porphyrin molecule.
2. The slow diffusion of bulky Co(II/III) complex into photoanode films along with the fast recombination of photo-excited electrons with the oxidized redox species make Co(II/III) complex electrolytes a challenging. Thus, facile engineering the porphyrin donor structure to make a favorable interaction with cobalt complexes, consequently a fast regeneration processes is necessary.
3. The volatile liquid electrolyte now frequently used in DSSC can not fulfil the request for the future application of DSSC because of its poor long-term stability under high temperature. Solid-state DSSC intrinsically own better stability. However, the PCE of solid state DSSC is much lower than their liquid based counterpart. As one kind of sensitizer owning intensive absorption ability, porphyrins are promising sensitizers for thin-film ss-DSSC. However, porphyrin based ss-DSSC exhibited lower PCE comparing to metal-free organic dyes. Thus, researchers might need a re-build of porphyrin structure to have a better contact with solid state hole transporting material (such as spiro-OMeTAD, CuSCN *etc*).
4. The last point is the lack of absorption ability around the range between 500 and 600 nm for typical porphyrins, which is the most intense area of solar photon-flux spectrum. Recently, some porphyrins featured with split B band were supposed to be beneficial to implement this short-coming of porphyrin sensitizer. However, the absorption valley between the B and Q bands are still obvious. Thus, further molecular engineering should be focused on implementing this natural character of porphyrins.

## Author contributions

MW and JL post the idea. JL, SL, and MW wrote the manuscript. JL and SL draw the figures.

### Conflict of interest statement

The authors declare that the research was conducted in the absence of any commercial or financial relationships that could be construed as a potential conflict of interest.

## References

[B1] Ahmad S. Guillén E. Kavan L. Grätzel M. Nazeeruddin M. K. (2013). Metal free sensitizer and catalyst for dye sensitized solar cells. Energy Environ. Sci. 6, 3439–3466. 10.1039/c3ee41888j

[B2] Ali N. Hussain A. Ahmed R. Wang M. Zhao C. Haq B. U. (2016). Advances in nanostructured thin film materials for solar cell applications. Renew. Sustain. Energy Rev. 59, 726–737. 10.1016/j.rser.2015.12.268

[B3] Ambre R. Chen K.-B. Yao C.-F. Luo L. Diau E. W.-G. Hung C.-H. (2012). Effects of porphyrinic meso-substituents on the photovoltaic performance of dye-sensitized solar cells: number and position of p-carboxyphenyl and thienyl groups on zinc porphyrins. J. Phys. Chem. C 116, 11907–11916. 10.1021/jp302145x

[B4] Ambre R. B. Chang G. F. Zanwar M. R. Yao C. F. Diau E. W. G. Hung C. H. (2013). New dual donor–acceptor (2D-π-2A) porphyrin sensitizers for stable and cost-effective dye-sensitized solar cells. Chem. Asian J. 8, 2144–2153. 10.1002/asia.201300328 23825005

[B5] Angaridis P. A. Lazarides T. Coutsolelos A. C. (2014). Functionalized porphyrin derivatives for solar energy conversion. Polyhedron 82, 19–32. 10.1016/j.poly.2014.04.039

[B6] Arja K. Elgland M. Nilsson K. P. R. (2018). Synthesis and characterization of oligothiophene–porphyrin-based molecules that can be utilized for optical assignment of aggregated amyloid-β morphotypes. Front. Chem. 6:391. 10.3389/fchem.2018.00391 30234103 PMC6129614

[B7] Badhulika S. Terse-Thakoor T. Chaves Villarreal C. Mulchandani A. (2015). Graphene hybrids: synthesis strategies and applications in sensors and sensitized solar cells. Front. Chem. 3:38. 10.3389/fchem.2015.00038 26176007 PMC4485227

[B8] Bai Y. Yang B. Chen X. Wang F. Hayat T. Alsaedi A.. (2018). Constructing desired vertical component distribution within a PBDB-T:ITIC-M photoactive layer via fine-tuning the surface free energy of a titanium chelate cathode buffer layer. Front. Chem. 6:292. 10.3389/fchem.2018.00292 30177964 PMC6109755

[B9] Barea E. M. Gónzalez-Pedro V. Ripollés-Sanchis T. Wu H.-P. Li L.-L. Yeh C.-Y. (2011). Porphyrin dyes with high injection and low recombination for highly efficient mesoscopic dye-sensitized solar cells. J. Phys. Chem. C 115, 10898–10902. 10.1021/jp2018378

[B10] Bella F. Gerbaldi C. Barolo C. Grätzel M. (2015). Aqueous dye-sensitized solar cells. Chem. Soc. Rev. 44, 3431–3473. 10.1039/C4CS00456F 25864577

[B11] Bessho T. Zakeeruddin S. M. Yeh C. Y. Diau E. W. G. Grätzel M. (2010). Highly efficient mesoscopic dye-sensitized solar cells based on donor–acceptor-substituted porphyrins. Angew. Chemie Int. Ed. 49, 6646–6649. 10.1002/anie.201002118 20687058

[B12] Cabau L. Kumar C. V. Moncho A. Clifford J. N. López N. Palomares E. (2015). A single atom change “switches-on” the solar-to-energy conversion efficiency of Zn-porphyrin based dye sensitized solar cells to 10.5%. Energy Environ. Sci. 8, 1368–1375. 10.1039/C4EE03320E

[B13] Campbell W. M. Burrell A. K. Officer D. L. Jolley K. W. (2004). Porphyrins as light harvesters in the dye-sensitised TiO 2 solar cell. Coord. Chem. Rev. 248, 1363–1379. 10.1016/j.ccr.2004.01.007

[B14] Campbell W. M. Jolley K. W. Wagner P. Wagner K. Walsh P. J. Gordon K. C. (2007). Highly efficient porphyrin sensitizers for dye-sensitized solar cells. J. Phys. Chem. C 111, 11760–11762. 10.1021/jp0750598

[B15] Cao K. Lu J. Cui J. Shen Y. Chen W. Alemu G. (2014). Highly efficient light harvesting ruthenium sensitizers for dye-sensitized solar cells featuring triphenylamine donor antennas. J. Mater. Chem. A 2, 4945–4953. 10.1039/C3TA15134D

[B16] Cao K. Wang M. (2013). Recent developments in sensitizers for mesoporous sensitized solar cells. Front. Optoelectr. 6, 373–385. 10.1007/s12200-013-0347-5

[B17] Cao Y. Saygili Y. Ummadisingu A. Teuscher J. Luo J. Pellet N.. (2017). 11% efficiency solid-state dye-sensitized solar cells with copper(II/I) hole transport materials. Nat. Commun. 8:15390. 10.1038/ncomms15390 28598436 PMC5472710

[B18] Chae S. H. Yoo K. Lee Y. S. Cho M. J. Kim J. H. Ko M. J. (2014). Novel π-extended porphyrin derivatives for use in dye-sensitized solar cells. J. Porphyr. Phthalocyanines 18, 569–578. 10.1142/S1088424614500308

[B19] Chang Y.-C. Wang C.-L. Pan T.-Y. Hong S.-H. Lan C.-M. Kuo H.-H.. (2011). A strategy to design highly efficient porphyrin sensitizers for dye-sensitized solar cells. Chem. Commun. 47, 8910–8912. 10.1039/c1cc12764k 21677971

[B20] Chen C.-Y. Wang M. Li J.-Y. Pootrakulchote N. Alibabaei L. Ngoc-le C.-H.. (2009). Highly efficient light-harvesting ruthenium sensitizer for thin-film dye-sensitized solar cells. ACS Nano 3, 3103–3109. 10.1021/nn900756s 19746929

[B21] Chen J. Liu Q. Li H. Zhao Z. Lu Z. Huang Y. (2018). Density functional theory investigations of D-A-D' structural molecules as donor materials in organic solar cell. Front. Chem. 6:200 10.3389/fchem.2018.00200 29915784 PMC5994543

[B22] Cherian S. Wamser C. C. (2000). Adsorption and Photoactivity of Tetra(4-carboxyphenyl)porphyrin (TCPP) on Nanoparticulate TiO2. J. Phys. Chem. B 104, 3624–3629. 10.1021/jp994459v

[B23] Cho Y. Soufiani A. M. Yun J. S. Kim J. Lee D. S. Seidel J. (2018). Mixed 3D−2D passivation treatment for mixed-cation lead mixed-halide perovskite solar cells for higher efficiency and better stability. Adv. Energy Mater. 8:1703392 10.29363/nanoge.hopv.2018.080

[B24] Chou H.-H. Reddy K. S. K. Wu H.-P. Guo B.-C. Lee H.-W. Diau E. W.-G.. (2016). Influence of phenylethynylene of push–pull zinc porphyrins on the photovoltaic performance. ACS Appl. Mater. Interfaces 8, 3418–3427. 10.1021/acsami.5b11554 26752243

[B25] Fakharuddin A. Jose R. Brown T. M. Fabregat-Santiago F. Bisquert J. (2014). A perspective on the production of dye-sensitized solar modules. Energy Environ. Sci. 7, 3952–3981. 10.1039/C4EE01724B

[B26] Fan S. Lv K. Sun H. Zhou G. Wang Z.-S. (2015). The position effect of electron-deficient quinoxaline moiety in porphyrin based sensitizers. J. Power Sources 279, 36–47. 10.1016/j.jpowsour.2014.12.143

[B27] Favereau L. Warnan J. Anne F. B. Pellegrin Y. Blart E. Jacquemin D. (2013). Diketopyrrolopyrrole-zinc porphyrin, a tuned panchromatic association for dye-sensitized solar cells. J. Mater. Chem. A 1, 7572–7575. 10.1039/c3ta11380a

[B28] Fournier M. Hoogeveen D. A. Bonke S. A. Spiccia L. Simonov Alexandr N. (2018). Cooperative silanetriolate-carboxylate sensitiser anchoring for outstanding stability and improved performance of dye-sensitised photoelectrodes. Sustain. Energy Fuels 2, 1707–1718. 10.1039/C8SE00056E

[B29] Freitag M. Teuscher J. Saygili Y. Zhang X. Giordano F. Liska P. (2017). Dye-sensitized solar cells for efficient power generation under ambient lighting. Nat. Photonics 11:372 10.1038/nphoton.2017.60

[B30] Galliano S. Bella F. Gerbaldi C. Falco M. Viscardi G. Grätzel M. (2017). Photoanode/electrolyte interface stability in aqueous dye-sensitized solar cells. Energy Technol. 5, 300–311. 10.1002/ente.201600285

[B31] Gao F. Wang Y. Shi D. Zhang J. Wang M. Jing X.. (2008). Enhance the optical absorptivity of nanocrystalline TiO2 film with high molar extinction coefficient ruthenium sensitizers for high performance dye-sensitized solar cells. J. Am. Chem. Soc. 130, 10720–10728. 10.1021/ja801942j 18642907

[B32] Griffith M. J. Sunahara K. Wagner P. Wagner K. Wallace G. G. Officer D. L.. (2012). Porphyrins for dye-sensitised solar cells: new insights into efficiency-determining electron transfer steps. Chem. Commun. 48, 4145–4162. 10.1039/c2cc30677h 22441329

[B33] Guo F. L. Li Z. Q. Liu X. P. Zhou L. Kong F. T. Chen W. C. (2016). Metal-free sensitizers containing hydantoin acceptor as high performance anchoring group for dye-sensitized solar cells. Adv. Funct. Mater. 26, 5733–5740. 10.1002/adfm.201601305

[B34] Hamann T. W. Jensen R. A. Martinson A. B. Van Ryswyk H. Hupp J. T. (2008). Advancing beyond current generation dye-sensitized solar cells. Energy Environ. Sci. 1, 66–78. 10.1039/b809672d

[B35] Hardin B. E. Snaith H. J. McGehee M. D. (2012). The renaissance of dye-sensitized solar cells. Nat. Photonics 6:162 10.1038/nphoton.2012.22

[B36] Hayashi H. Higashino T. Kinjo Y. Fujimori Y. Kurotobi K. Chabera P.. (2015). Effects of immersion solvent on photovoltaic and photophysical properties of porphyrin-sensitized solar cells. ACS Appl. Mater. Interfaces 7, 18689–18696. 10.1021/acsami.5b05163 26266818

[B37] He H. Gurung A. Si L. (2012a). 8-Hydroxylquinoline as a strong alternative anchoring group for porphyrin-sensitized solar cells. Chem. Commun. 48, 5910–5912. 10.1039/c2cc31440a 22573243

[B38] He H. Gurung A. Si L. Sykes A. G. (2012b). A simple acrylic acid functionalized zinc porphyrin for cost-effective dye-sensitized solar cells. Chem. Commun. 48, 7619–7621. 10.1039/c2cc33337f 22733032

[B39] Heiniger L.-P. O'Brien P. G. Soheilnia N. Yang Y. Kherani N. P. Grätzel M.. (2013). See-through dye-sensitized solar cells: photonic reflectors for tandem and building integrated photovoltaics. Adv. Mater. 25, 5734–5741. 10.1002/adma.201302113 23966106

[B40] Higashino T. Imahori H. (2015). Porphyrins as excellent dyes for dye-sensitized solar cells: recent developments and insights. Dalton Trans. 44, 448–463. 10.1039/C4DT02756F 25381701

[B41] Huang D. Lu J. Li S. Luo Y. Zhao C. Hu B.. (2014). Fabrication of cobalt porphyrin. Electrochemically reduced graphene oxide hybrid Films for electrocatalytic hydrogen evolution in aqueous solution. Langmuir 30, 6990–6998. 10.1021/la501052m 24856539

[B42] Huang F. Pascoe A. R. Wu W. Q. Ku Z. Peng Y. Zhong J.. (2017). Effect of the microstructure of the functional layers on the efficiency of perovskite solar cells. Adv. Mater. 29:1601715. 10.1002/adma.201601715 28225146

[B43] Imahori H. Kang S. Hayashi H. Haruta M. Kurata H. Isoda S.. (2010). Photoinduced charge carrier dynamics of Zn-porphyrin-TiO_2_ electrodes: the key role of charge recombination for solar cell performance. J. Phys. Chem. A 115, 3679–3690. 10.1021/jp103747t 20961148

[B44] Imahori H. Umeyama T. Ito S. (2009). Large π-aromatic molecules as potential sensitizers for highly efficient dye-sensitized solar cells. Acc. Chem. Res. 42, 1809–1818. 10.1021/ar900034t 19408942

[B45] Jiao C. Zu N. Huang K.-W. Wang P. Wu J. (2011). Perylene anhydride fused porphyrins as near-infrared sensitizers for dye-sensitized solar cells. Org. Lett. 13, 3652–3655. 10.1021/ol201303h 21699168

[B46] Jradi F. M. O'Neil D. Kang X. Wong J. Szymanski P. Parker T. C. (2015). A step toward efficient panchromatic multi-chromophoric sensitizers for dye sensitized solar cells. Chem. Mater. 27, 6305–6313. 10.1021/acs.chemmater.5b02006

[B47] Kakiage K. Aoyama Y. Yano T. Oya K. Fujisawa J.-,i. Hanaya M. (2015). Highly-efficient dye-sensitized solar cells with collaborative sensitization by silyl-anchor and carboxy-anchor dyes. Chem. Commun. 51, 15894–15897. 10.1039/C5CC06759F 26393334

[B48] Kang M. S. Choi I. T. Kim Y. W. You B. S. Kang S. H. Hong J. Y. (2013). Novel D–π-A structured Zn (ii)–porphyrin dyes with bulky fluorenyl substituted electron donor moieties for dye-sensitized solar cells. J. Mater. Chem. A 1, 9848–9852. 10.1039/c3ta11818e

[B49] Kang M. S. Kang S. H. Kim S. G. Choi I. T. Ryu J. H. Ju M. J.. (2012). Novel D–π-A structured Zn (II)-porphyrin dyes containing a bis (3, 3-dimethylfluorenyl) amine moiety for dye-sensitised solar cells. Chem. Commun. 48, 9349–9351. 10.1039/c2cc31384g 22499080

[B50] Kay A. Graetzel M. (1993). Artificial photosynthesis. 1. Photosensitization of titania solar cells with chlorophyll derivatives and related natural porphyrins. J. Phys. Chem. 97, 6272–6277. 10.1021/j100125a029

[B51] Kesters J. Verstappen P. Kelchtermans M. Lutsen L. Vanderzande D. Maes W. (2015). Porphyrin-based bulk heterojunction organic photovoltaics: the rise of the colors of life. Adv. Energy Mater. 5:1500218 10.1002/aenm.201500218

[B52] Ku Z. Li X. Liu G. Wang H. Rong Y. Xu M. (2013). Transparent NiS counter electrodes for thiolate/disulfide mediated dye-sensitized solar cells. J. Mater. Chem. A 1, 237–240. 10.1039/C2TA00304J

[B53] Kurotobi K. Toude Y. Kawamoto K. Fujimori Y. Ito S. Chabera P.. (2013). Highly asymmetrical porphyrins with enhanced push–pull character for dye-sensitized solar cells. Chem. Eur. J. 19, 17075–17081. 10.1002/chem.201303460 24227165

[B54] Ladomenou K. Kitsopoulos T. Sharma G. Coutsolelos A. (2014). The importance of various anchoring groups attached on porphyrins as potential dyes for DSSC applications. RSC Adv. 4, 21379–21404. 10.1039/C4RA00985A

[B55] Lan C.-M. Wu H.-P. Pan T.-Y. Chang C.-W. Chao W.-S. Chen C.-T. (2012). Enhanced photovoltaic performance with co-sensitization of porphyrin and an organic dye in dye-sensitized solar cells. Energy Environ. Sci. 5, 6460–6464. 10.1039/c2ee21104a

[B56] Lee C.-W. Lu H.-P. Reddy N. M. Lee H.-W. Diau E. W.-G. Yeh C.-Y. (2011). Electronically coupled porphyrin-arene dyads for dye-sensitized solar cells. Dyes Pigments 91, 317–323. 10.1016/j.dyepig.2011.04.010

[B57] Lee C. W. Lu H. P. Lan C. M. Huang Y. L. Liang Y. R. Yen W. N.. (2009). Novel Zinc Porphyrin Sensitizers for Dye-Sensitized Solar Cells: Synthesis and Spectral, Electrochemical, and photovoltaic properties. Chem. Eur. J. 15, 1403–1412. 10.1002/chem.200801572 19097125

[B58] Li L.-L. Diau E. W.-G. (2013). Porphyrin-sensitized solar cells. Chem. Soc. Rev. 42, 291–304. 10.1039/C2CS35257E 23023240

[B59] Li W. Liu Z. Wu H. Cheng Y.-B. Zhao Z. He H. (2015). Thiophene-functionalized porphyrins: synthesis, photophysical properties, and photovoltaic performance in dye-sensitized solar cells. J. Phys. Chem. C 119, 5265–5273. 10.1021/jp509842p

[B60] Li W. Liu Z. Xu X. Cheng Y.-B. Zhao Z. He H. (2014a). Near-infrared absorbing porphyrin dyes with perpendicularly extended π-conjugation for dye-sensitized solar cells. RSC Adv. 4, 50897–50905. 10.1039/C4RA08338E

[B61] Li W. Si L. Liu Z. Wu H. Zhao Z. Cheng Y.-B. (2014b). Bis (9, 9-dihexyl-9H-fluorene-7-yl) amine (BDFA) as a new donor for porphyrin-sensitized solar cells. Org. Electron. 15, 2448–2460. 10.1016/j.orgel.2014.07.006

[B62] Li W. Si L. Liu Z. Zhao Z. He H. Zhu K. (2014c). Fluorene functionalized porphyrins as broadband absorbers for TiO_2_ nanocrystalline solar cells. J. Mater. Chem. A 2, 13667–13674. 10.1039/C4TA01954G

[B63] Lin C.-Y. Wang Y.-C. Hsu S.-J. Lo C.-F. Diau E. W.-G. (2009). Preparation and spectral, electrochemical, and photovoltaic properties of acene-modified zinc porphyrins. J. Phys. Chem. C 114, 687–693. 10.1021/jp909232b

[B64] Lin K. Wang S. Wang Z. Yin Q. Liu X. Jia J.. (2018). Electron acceptors with a truxene core and perylene diimide branches for organic solar cells: the effect of ring-fusion. Front. Chem. 6:328. 10.3389/fchem.2018.00328 30234096 PMC6131300

[B65] Liu Y. Lin H. Dy J. T. Tamaki K. Nakazaki J. Nakayama D.. (2011). N-fused carbazole–zinc porphyrin–free-base porphyrin triad for efficient near-IR dye-sensitized solar cells. Chem. Commun. 47, 4010–4012. 10.1039/c0cc03306e 21290072

[B66] Liu Y. Lin H. Li J. Dy J. T. Tamaki K. Nakazaki J.. (2012). Ethynyl-linked push–pull porphyrin hetero-dimers for near-IR dye-sensitized solar cells: photovoltaic performances versus excited-state dynamics. Phys. Chem. Chem. Phys. 14, 16703–16712. 10.1039/c2cp43165c 23138644

[B67] Liu Y. Xiang N. Feng X. Shen P. Zhou W. Weng C. (2009). Thiophene-linked porphyrin derivatives for dye-sensitized solar cells. Chem. Commun. 18, 2499–2501. 10.1039/b821985k 19532869

[B68] Lo C.-F. Hsu S.-J. Wang C.-L. Cheng Y.-H. Lu H.-P. Diau E. W.-G. (2010). Tuning spectral and electrochemical properties of porphyrin-sensitized solar cells. J. Phys. Chem. C 114, 12018–12023. 10.1021/jp103561c

[B69] Lu F. Feng Y. Wang X. Zhao Y. Yang G. Zhang J. (2017). Influence of the additional electron-withdrawing unit in β-functionalized porphyrin sensitizers on the photovoltaic performance of dye-sensitized solar cells. Dyes Pigments 139, 255–263. 10.1016/j.dyepig.2016.12.027

[B70] Lu H. P. Tsai C. Y. Yen W. N. Hsieh C. P. Lee C. W. Yeh C. Y. (2009). Control of dye aggregation and electron injection for highly efficient porphyrin sensitizers adsorbed on semiconductor films with varying ratios of coadsorbate. J. Phys. Chem. C 113, 20990–20997. 10.1021/jp908100v

[B71] Lu J. Chang Y. C. Cheng H. Y. Wu H. P. Cheng Y. Wang M.. (2015a). Molecular engineering of organic dyes with a hole-extending donor tail for efficient all-solid-state dye-sensitized solar cells. ChemSusChem 8, 2529–2536. 10.1002/cssc.201500309 26119886

[B72] Lu J. Jiang L. Li W. Li F. Pai N. K. Scully A. D. (2017). Diammonium and monoammonium mixed-organic-cation perovskites for high performance solar cells with improved stability. Adv. Energy Mater. 7:1700444 10.1002/aenm.201700444

[B73] Lu J. Li H. Liu S. Chang Y.-C. Wu H.-P. Cheng Y.. (2016). Novel porphyrin-preparation, characterization, and applications in solar energy conversion. Phys. Chem. Chem. Phys. 18, 6885–6892. 10.1039/C5CP05658F 26878900

[B74] Lu J. Lin X. Jiao X. Gengenbach T. Scully A. D. Jiang L. (2018). Interfacial benzenethiol modification facilitates charge transfer and improves stability of cm-sized metal halide perovskite solar cells with up to 20% efficiency. Energy Environ. Sci. 11, 1880–1889. 10.1039/C8EE00754C

[B75] Lu J. Liu S. Li H. Shen Y. Xu J. Cheng Y. (2014a). Pyrene-conjugated porphyrins for efficient mesoscopic solar cells: the role of the spacer. J. Mater. Chem. A 2, 17495–17501. 10.1039/C4TA03435J

[B76] Lu J. Liu S. Shen Y. Xu J. Cheng Y. Wang M. (2015b). Alkyl-thiophene functionalized D-π-a porphyrins for mesoscopic solar cells. Electrochim. Acta 179, 187–196. 10.1016/j.electacta.2015.02.194

[B77] Lu J. Xu X. Cao K. Cui J. Zhang Y. Shen Y. (2013a). D–π-A structured porphyrins for efficient dye-sensitized solar cells. J. Mater. Chem. A 1, 10008–10015. 10.1039/c3ta11870c

[B78] Lu J. Xu X. Li Z. Cao K. Cui J. Zhang Y.. (2013b). Zinc porphyrins with a pyridine-ring-anchoring group for dye-sensitized solar cells. Chem. Asian J. 8, 956–962. 10.1002/asia.201201136 23424179

[B79] Lu J. Zhang B. Liu S. Li H. Yuan H. Shen Y.. (2014b). A cyclopenta [1, 2-b: 5, 4-b′] dithiophene–porphyrin conjugate for mesoscopic solar cells: a D–π-D–A approach. Phys. Chem. Chem. Phys. 16, 24755–24762. 10.1039/C4CP03425B 25315179

[B80] Lu J. Zhang B. Yuan H. Xu X. Cao K. Cui J. (2014c). D– π-A porphyrin sensitizers with π-extended conjugation for mesoscopic solar cells. J. Phys. Chem. C 118, 14739–14748. 10.1021/jp5014829

[B81] Lu J.-F. Liu Z. Pai N. Jiang L. Bach U. Simonov A. N. (2018). molecular engineering of zinc-porphyrin sensitisers for p-type dye-sensitised solar cells. ChemPlusChem 83, 711–720. 10.1002/cplu.201800104 31950629

[B82] Luo J. Xu M. Li R. Huang K.-W. Jiang C. Qi Q.. (2013). N-annulated perylene as an efficient electron donor for porphyrin-based dyes: enhanced light-harvesting ability and high-efficiency Co (II/III)-based dye-sensitized solar cells. J. Am. Chem. Soc. 136, 265–272. 10.1021/ja409291g 24345083

[B83] Luo J. Zhang J. Huang K.-W. Qi Q. Dong S. Zhang J. (2016). N-Annulated perylene substituted zinc–porphyrins with different linking modes and electron acceptors for dye sensitized solar cells. J. Mater. Chem. A 4, 8428–8434. 10.1039/C6TA02509A

[B84] Luo L. Ambre R. B. Mane S. B. Diau E. W.-G. Hung C.-H. (2015). The cis-isomer performs better than the trans-isomer in porphyrin-sensitized solar cells: interfacial electron transport and charge recombination investigations. Phys. Chem. Chem. Phys. 17, 20134–20143. 10.1039/C5CP02367J 26174451

[B85] Mai C.-L. Huang W.-K. Lu H.-P. Lee C.-W. Chiu C.-L. Liang Y.-R.. (2010). Synthesis and characterization of diporphyrin sensitizers for dye-sensitized solar cells. Chem. Commun. 46, 809–811. 10.1039/B917316A 20087528

[B86] Mai C. L. Moehl T. Hsieh C. H. Décoppet J.-D. Zakeeruddin S. M. Grätzel M.. (2015). Porphyrin sensitizers bearing a pyridine-type anchoring group for dye-sensitized solar cells. ACS Appl. Mater. Interfaces 7, 14975–14982. 10.1021/acsami.5b03783 26083949

[B87] Marszalek M. Arendse F. D. Decoppet J. D. Babkair S. S. Ansari A. A. Habib S. S. (2014). Ionic liquid–sulfolane composite electrolytes for high-performance and stable dye-sensitized solar cells. Adv. Energy Mater. 4:1301235 10.1002/aenm.201301235

[B88] Martin C. Ziółek M. Douhal A. (2016). Ultrafast and fast charge separation processes in real dye-sensitized solar cells. J. Photochem. Photobiol. C Photochem. Rev. 26, 1–30. 10.1016/j.jphotochemrev.2015.12.001

[B89] Mathew S. Iijima H. Toude Y. Umeyama T. Matano Y. Ito S. (2011). Optical, electrochemical, and photovoltaic effects of an electron-withdrawing tetrafluorophenylene bridge in a push–pull porphyrin sensitizer used for dye-sensitized solar cells. J. Phys. Chem. C 115, 14415–14424. 10.1021/jp2030208

[B90] Mathew S. Yella A. Gao P. Humphry-Baker R. Curchod B. F. Ashari-Astani N.. (2014). Dye-sensitized solar cells with 13% efficiency achieved through the molecular engineering of porphyrin sensitizers. Nat. Chem. 6, 242–247. 10.1038/nchem.1861 24557140

[B91] Obraztsov I. Kutner W. D'Souza F. (2017). Evolution of molecular design of porphyrin chromophores for photovoltaic materials of superior light-to-electricity conversion efficiency. Solar RRL 1, 1600002 10.1002/solr.201600002

[B92] Ooyama Y. Inoue S. Nagano T. Kushimoto K. Ohshita J. Imae I. (2011). Dye-sensitized solar cells based on donor–acceptor π-conjugated fluorescent dyes with a pyridine ring as an electron-withdrawing anchoring group. Angew. Chemie 123, 7567–7571. 10.1002/ange.201102552 21717551

[B93] Ooyama Y. Uenaka K. Kamimura T. Ozako S. Kanda M. Koide T. (2016). Dye-sensitized solar cell based on an inclusion complex of a cyclic porphyrin dimer bearing four 4-pyridyl groups and fullerene C 60. RSC Adv. 6, 16150–16158. 10.1039/C6RA01131D

[B94] O'Regan B. Xiaoe L. Ghaddar T. (2012). Dye adsorption, desorption, and distribution in mesoporous TiO 2 films, and its effects on recombination losses in dye sensitized solar cells. Energy Environ. Sci. 5, 7203–7215. 10.1039/c2ee21341a

[B95] Pashaei B. Shahroosvand H. Graetzel M. Nazeeruddin M. K. (2016). Influence of ancillary ligands in dye-sensitized solar cells. Chem. Rev. 116, 9485–9564. 10.1021/acs.chemrev.5b00621 27479482

[B96] Pellej A. L. Kumar C. V. Clifford J. N. Palomares E. (2014). D-π-A porphyrin employing an indoline donor group for high efficiency dye-sensitized solar cells. J. Phys. Chem. C 118, 16504–16509. 10.1021/jp411715n

[B97] Qi Q. Li R. Luo J. Zheng B. Huang K.-W. Wang P. (2015). Push–pull type porphyrin based sensitizers: the effect of donor structure on the light-harvesting ability and photovoltaic performance. Dyes Pigments 122, 199–205. 10.1016/j.dyepig.2015.06.019

[B98] Qi X. Lo Y.-C. Zhao Y. Xuan L. Ting H.-C. Wong K.-T.. (2018). Two novel small molecule donors and the applications in bulk-heterojunction solar cells. Front. Chem. 6:260. 10.3389/fchem.2018.00260 30013968 PMC6036481

[B99] Ripolles-Sanchis T. Guo B.-C. Wu H.-P. Pan T.-Y. Lee H.-W. Raga S. R.. (2012). Design and characterization of alkoxy-wrapped push–pull porphyrins for dye-sensitized solar cells. Chem. Commun. 48, 4368–4370. 10.1039/c2cc31111a 22447157

[B100] Sakurada T. Arai Y. Segawa H. (2014). Porphyrins with β-acetylene-bridged functional groups for efficient dye-sensitized solar cells. RSC Adv. 4, 13201–13204. 10.1039/c3ra41317a

[B101] Seo K. D. Lee M. J. Song H. M. Kang H. S. Kim H. K. (2012). Novel D-π-A system based on zinc porphyrin dyes for dye-sensitized solar cells: synthesis, electrochemical, and photovoltaic properties. Dyes Pigments 94, 143–149. 10.1016/j.dyepig.2011.12.006

[B102] Sharma G. Daphnomili D. Angaridis P. A. Biswas S. Coutsolelos A. (2013). Effect of thiourea incorporation in the electrolyte on the photovoltaic performance of the DSSC sensitized with pyridyl functionalized porphyrin. Electrochim. Acta 102, 459–465. 10.1016/j.electacta.2013.04.003

[B103] Shiu J.-W. Chang Y.-C. Chan C.-Y. Wu H.-P. Hsu H.-Y. Wang C.-L. (2015). Panchromatic co-sensitization of porphyrin-sensitized solar cells to harvest near-infrared light beyond 900 nm. J. Mater. Chem. A 3, 1417–1420. 10.1039/C4TA06589A

[B104] Si L. He H. Zhu K. (2014). 8-Hydroxylquinoline-conjugated porphyrins as broadband light absorbers for dye-sensitized solar cells. N J. Chem. 38, 1565–1572. 10.1039/c3nj01643a

[B105] Sirithip K. Prachumrak N. Rattanawan R. Keawin T. Sudyoadsuk T. Namuangruk S.. (2015). Zinc–porphyrin dyes with different meso-aryl substituents for dye-sensitized solar cells: experimental and theoretical studies. Chem. Asian J. 10, 882–893. 10.1002/asia.201402766 25267373

[B106] Song H. Liu Q. Xie Y. (2018). Porphyrin-sensitized solar cells: systematic molecular optimization, coadsorption and cosensitization. Chem. Commun. 54, 1811–1824. 10.1039/C7CC09671B 29372729

[B107] Sreenivasu M. Suzuki A. Adachi M. Kumar C. V. Srikanth B. Rajendar S.. (2014). Synthesis and characterization of donor–π-acceptor-based porphyrin sensitizers: potential application of dye-sensitized solar cells. Chem. Eur. J. 20, 14074–14083. 10.1002/chem.201403660 25210010

[B108] Sun X. Wang Y. Li X. Ågren H. Zhu W. Tian H.. (2014). Cosensitizers for simultaneous filling up of both absorption valleys of porphyrins: a novel approach for developing efficient panchromatic dye-sensitized solar cells. Chem. Commun. 50, 15609–15612. 10.1039/C4CC07963A 25358496

[B109] Tang Y. Wang Y. Li X. Ågren H. Zhu W.-H. Xie Y. (2015). Porphyrins containing a triphenylamine donor and up to eight alkoxy chains for dye-sensitized solar cells: a high efficiency of 10.9%. ACS Appl. Mater. Interfaces 7, 27976–27985. 10.1021/acsami.5b10624 26606858

[B110] Urbani M. Grätzel M. Nazeeruddin M. K. Torres T. (2014). Meso-substituted porphyrins for dye-sensitized solar cells. Chem. Rev. 114, 12330–12396. 10.1021/cr5001964 25495339

[B111] van der Salm H. Lind S. J. Griffith M. J. Wagner P. Wallace G. G. Officer D. L. (2015). Probing donor–acceptor interactions in meso-substituted Zn (II) porphyrins using resonance raman spectroscopy and computational chemistry. J. Phys. Chem. C 119, 22379–22391. 10.1021/acs.jpcc.5b07129

[B112] Vittal R. Ho K.-C. (2017). Zinc oxide based dye-sensitized solar cells: a review. Renew. Sustain. Energy Rev. 70, 920–935 10.1016/j.rser.2016.11.273

[B113] Wang B. Fu Y. Yan C. Zhang R. Yang Q. Han Y.. (2018). Insight into the role of PC71BM on enhancing the photovoltaic performance of ternary organic solar cells. Front. Chem. 6:198. 10.3389/fchem.2018.00198 29922645 PMC5996040

[B114] Wang C.-L. Chang Y.-C. Lan C.-M. Lo C.-F. Diau E. W.-G. Lin C.-Y. (2011). Enhanced light harvesting with π-conjugated cyclic aromatic hydrocarbons for porphyrin-sensitized solar cells. Energy Environ. Sci. 4, 1788–1795. 10.1039/c0ee00767f

[B115] Wang C.-L. Hu J.-Y. Wu C.-H. Kuo H.-H. Chang Y.-C. Lan Z.-J. (2014a). Highly efficient porphyrin-sensitized solar cells with enhanced light harvesting ability beyond 800 nm and efficiency exceeding 10%. Energy Environ. Sci. 7, 1392–1396. 10.1039/c3ee44168g

[B116] Wang C.-L. Lan C.-M. Hong S.-H. Wang Y.-F. Pan T.-Y. Chang C.-W. (2012). Enveloping porphyrins for efficient dye-sensitized solar cells. Energy Environ. Sci. 5, 6933–6940. 10.1039/c2ee03308a

[B117] Wang C.-L. Shiu J.-W. Hsiao Y.-N. Chao P.-S. Wei-Guang Diau E. Lin C.-Y. (2014b). Co-sensitization of zinc and free-base porphyrins with an organic dye for efficient dye-sensitized solar cells. J. Phys. Chem. C 118, 27801–27807. 10.1021/jp510057b

[B118] Wang C.-L. Zhang M. Hsiao Y.-H. Tseng C.-K. Liu C.-L. Xu M. (2016). Porphyrins bearing a consolidated anthryl donor with dual functions for efficient dye-sensitized solar cells. Energy Environ. Sci. 9, 200–206. 10.1039/C5EE02505B

[B119] Wang M. Anghel A. M. Marsan B. Cevey Ha N.-L. Pootrakulchote N. Zakeeruddin S. M.. (2009). CoS supersedes Pt as efficient electrocatalyst for triiodide reduction in dye-sensitized solar cells. J. Am. Chem. Soc. 131, 15976–15977. 10.1021/ja905970y 19845335

[B120] Wang M. Bai J. Le Formal F. Moon S.-J. Cevey-Ha L. Humphry-Baker R. (2012a). Solid-state dye-sensitized solar cells using ordered TiO2 nanorods on transparent conductive oxide as photoanodes. J. Phys. Chem. C 116, 3266–3273. 10.1021/jp209130x

[B121] Wang M. Chamberland N. Breau L. Moser J.-E. Humphry-Baker R. Marsan B.. (2010). An organic redox electrolyte to rival triiodide/iodide in dye-sensitized solar cells. Nat. Chem. 2:385. 10.1038/nchem.610 20414239

[B122] Wang M. Grätzel C. Zakeeruddin S. M. Grätzel M. (2012b). Recent developments in redox electrolytes for dye-sensitized solar cells. Energy Environ. Sci. 5, 9394–9405. 10.1039/c2ee23081j

[B123] Wang Q. Campbell W. M. Bonfantani E. E. Jolley K. W. Officer D. L. Walsh P. J.. (2005). Efficient light harvesting by using green Zn-porphyrin-sensitized nanocrystalline TiO_2_ films. J. Phys. Chem. B 109, 15397–15409. 10.1021/jp052877w 16852953

[B124] Wang Y. Chen B. Wu W. Li X. Zhu W. Tian H.. (2014c). Efficient solar cells sensitized by porphyrins with an extended conjugation framework and a carbazole donor: from molecular design to cosensitization. Angew. Chem. Int. Ed. 53, 10779–10783. 10.1002/anie.201406190 25132108

[B125] Wang Y. Xu L. Wei X. Li X. Ågren H. Wu W. (2014d). 2-Diphenylaminothiophene as the donor of porphyrin sensitizers for dye-sensitized solar cells. N. J. Chem. 38, 3227–3235. 10.1039/C4NJ00651H

[B126] Warnan J. Favereau L. Meslin F. Severac M. Blart E. Pellegrin Y.. (2012). Diketopyrrolopyrrole–porphyrin conjugates as broadly absorbing sensitizers for dye-sensitized solar cells. ChemSusChem 5, 1568–1577. 10.1002/cssc.201100764 22791585

[B127] Wu C.-H. Chen M.-C. Su P.-C. Kuo H.-H. Wang C.-L. Lu C.-Y. (2014). Porphyrins for efficient dye-sensitized solar cells covering the near-IR region. J. Mater. Chem. A 2, 991–999. 10.1039/C3TA14208F

[B128] Wu H.-P. Lan C.-M. Hu J.-Y. Huang W.-K. Shiu J.-W. Lan Z.-J.. (2013). Hybrid titania photoanodes with a nanostructured multi-layer configuration for highly efficient dye-sensitized solar cells. J. Phys. Chem. Lett. 4, 1570–1577. 10.1021/jz400620q 26282315

[B129] Wu H.-P. Ou Z.-W. Pan T.-Y. Lan C.-M. Huang W.-K. Lee H.-W. (2012). Molecular engineering of cocktail co-sensitization for efficient panchromatic porphyrin-sensitized solar cells. Energy Environ. Sci. 5, 9843–9848. 10.1039/c2ee22870j

[B130] Wu J. Lan Z. Lin J. Huang M. Huang Y. Fan L.. (2017). Counter electrodes in dye-sensitized solar cells. Chem. Soc. Rev. 46, 5975–6023. 10.1039/C6CS00752J 28840218

[B131] Wu S.-L. Lu H.-P. Yu H.-T. Chuang S.-H. Chiu C.-L. Lee C.-W. (2010). Design and characterization of porphyrin sensitizers with a push-pull framework for highly efficient dye-sensitized solar cells. Energy Environ. Sci. 3, 949–955. 10.1039/c003872p

[B132] Xiang W. Huang F. Cheng Y.-B. Bach U. Spiccia L. (2013). Aqueous dye-sensitized solar cell electrolytes based on the cobalt(ii)/(iii) tris(bipyridine) redox couple. Energy Environ. Sci. 6, 121–127. 10.1039/C2EE23317G

[B133] Xie R. Ying L. Liao H. Chen Z. Huang F. Cao Y. (2018). Efficient Non-fullerene Organic Solar Cells Enabled by Sequential Fluorination of Small-Molecule Electron Acceptors. Front. Chem. 6:303. 10.3389/fchem.2018.00303 30094231 PMC6071513

[B134] Xie Y. Tang Y. Wu W. Wang Y. Liu J. Li X.. (2015). Porphyrin cosensitization for a photovoltaic efficiency of 11.5%: a record for non-ruthenium solar cells based on iodine electrolyte. J. Am. Chem. Soc. 137, 14055–14058. 10.1021/jacs.5b09665 26492075

[B135] Xu X. Zhang H. Cao K. Cui J. Lu J. Zeng X.. (2014). Lead methylammonium triiodide perovskite-based solar cells: an interfacial charge-transfer investigation. ChemSusChem 7, 3088–3094. 10.1002/cssc.201402566 25213607

[B136] Yang J. Ganesan P. Teuscher J. Moehl T. Kim Y. J. Yi C.. (2014). Influence of the donor size in D– π-A organic dyes for dye-sensitized solar cells. J. Am. Chem. Soc. 136, 5722–5730. 10.1021/ja500280r 24655036

[B137] Yao Z. Zhang M. Li R. Yang L. Qiao Y. Wang P. (2015a). A metal-free N-annulated thienocyclopentaperylene dye: power conversion efficiency of 12% for dye-sensitized solar cells. Angew. Chem. Int. Ed. 127, 6092–6096. 10.1002/ange.201501195 25820975

[B138] Yao Z. Zhang M. Wu H. Yang L. Li R. Wang P. (2015b). Donor/acceptor indenoperylene dye for highly efficient organic dye-sensitized solar cells. J. Am. Chem. Soc. 137, 3799–3802. 10.1021/jacs.5b01537 25742441

[B139] Ye M. Wen X. Wang M. Iocozzia J. Zhang N. Lin C. (2015). Recent advances in dye-sensitized solar cells: from photoanodes, sensitizers and electrolytes to counter electrodes. Mater. Today 18, 155–162. 10.1016/j.mattod.2014.09.001

[B140] Ye S. Kathiravan A. Hayashi H. Tong Y. Infahsaeng Y. Chabera P. (2013). Role of adsorption structures of zn-porphyrin on TiO_2_ in dye-sensitized solar cells studied by sum frequency generation vibrational spectroscopy and ultrafast spectroscopy. J. Phys. Chem. C 117, 6066–6080. 10.1021/jp400336r

[B141] Yella A. Lee H.-W. Tsao H. N. Yi C. Chandiran A. K. Nazeeruddin M. K.. (2011). Porphyrin-sensitized solar cells with cobalt (II/III)–based redox electrolyte exceed 12 percent efficiency. Science 334, 629–634. 10.1126/science.1209688 22053043

[B142] Yella A. Mai C. L. Zakeeruddin S. M. Chang S. N. Hsieh C. H. Yeh C. Y. (2014). Molecular engineering of push–pull porphyrin dyes for highly efficient dye-sensitized solar cells: the role of benzene spacers. Angew. Chem. 126, 3017–3021. 10.1002/ange.201309343 24501108

[B143] Zhang J. Zhang J.-Z. Li H.-B. Wu Y. Geng Y. Su Z.-M. (2014). Rational modifications on champion porphyrin dye SM315 using different electron-withdrawing moieties toward high performance dye-sensitized solar cells. Phys. Chem. Chem. Phys. 16, 24994–25003. 10.1039/C4CP03355H 25327722

[B144] Zhang M.-D. Zhang Z.-Y. Bao Z.-Q. Ju Z.-M. Wang X.-Y. Zheng H.-G. (2014). Promising alkoxy-wrapped porphyrins with novel push–pull moieties for dye-sensitized solar cells. J. Mater. Chem. A 2, 14883–14889. 10.1039/C4TA02335H

[B145] Zhang T. Qian X. Zhang P.-F. Zhu Y.-Z. Zheng J.-Y. (2015). A meso–meso directly linked porphyrin dimer-based double D–π-A sensitizer for efficient dye-sensitized solar cells. Chem. Commun. 51, 3782–3785. 10.1039/C4CC09640A 25647634

[B146] Zhang X. Jia Y. Zhao D. Gou F. Gao H. Xu C. (2017). Substituted and anchoring groups improve the efficiency of dye-sensitized solar cells. Chem. Select 2, 4084–4091. 10.1002/slct.201700494

[B147] Zhao L. Wagner P. Barnsley J. E. Clarke T. M. Gordon K. C. Mori S.. (2016). Enhancement of dye regeneration kinetics in dichromophoric porphyrin–carbazole triphenylamine dyes influenced by more exposed radical cation orbitals. Chem. Sci. 7, 3506–3516. 10.1039/C6SC00429F 29997843 PMC6007200

[B148] Zhou H. Yang L. You W. (2012). Rational design of high performance conjugated polymers for organic solar cells. Macromolecules 45, 607–632. 10.1021/ma201648t

[B149] Zhou N. Prabakaran K. Lee B. Chang S. H. Harutyunyan B. Guo P.. (2015). Metal-free tetrathienoacene sensitizers for high-performance dye-sensitized solar cells. J. Am. Chem. Soc. 137, 4414–4423. 10.1021/ja513254z 25768124

