# Supplementary material for: Push-Pull Zinc Porphyrins as Light-Harvesters for Efficient Dye-Sensitized Solar Cells
Source: Front Chem. 2018 Nov 16;6:541. doi: 10.3389/fchem.2018.00541 (PMC6251255; doi:10.3389/fchem.2018.00541)
Supplement: Supplementary file 1 [file Data_Sheet_1.PDF]

Supporting information

Push-pull Zinc Porphyrins as Light-harvesters for Efficient Dye-Sensitized Solar Cells

Jianfeng Lu,[a,b] Shuangshuang Liu[a] and Mingkui Wang [a]*

[a] Wuhan National Laboratory for Optoelectronics, Huazhong University of Science and Technology, 1037 Luoyu Road, Wuhan 430074, P.R. China, E-mail: *mingkui.wang@mail.hust.edu.cn*

[b] School of Chemistry, Monash University, Melbourne, Victoria, 3800, Australia.

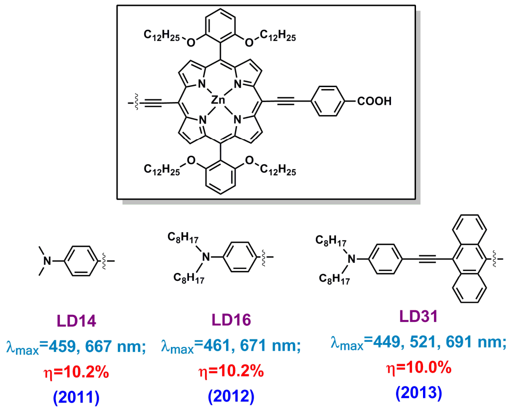

Fig. S1 Structure of some porphyrins bearing a dialkylaminophenyl donor group.

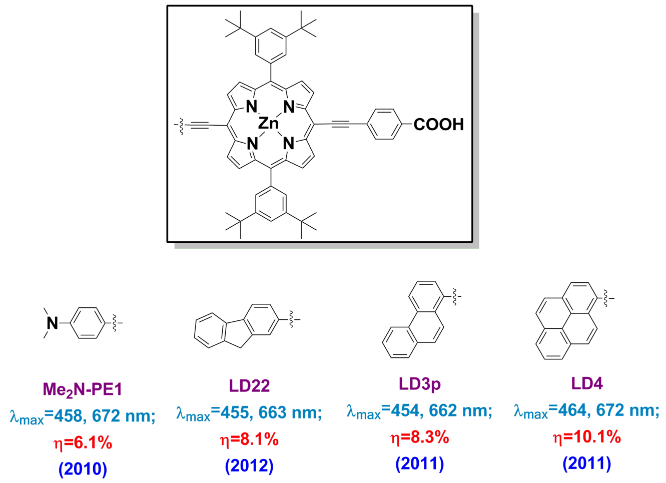

Fig. S2 Structure of porphyrins bearing with benzene and derivatives as donor group.

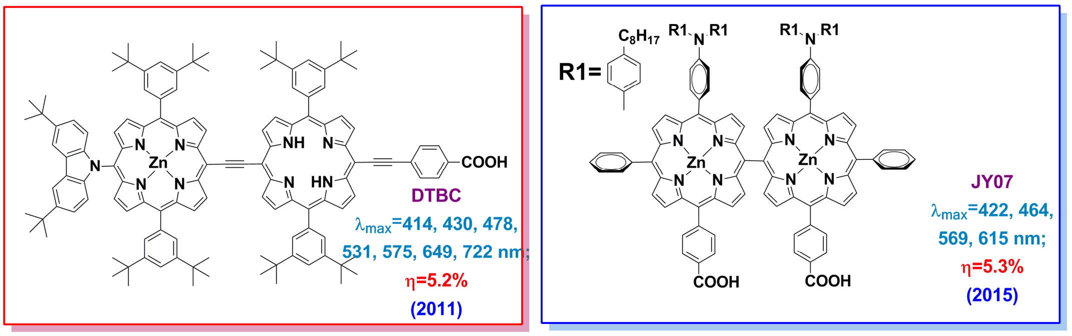

Fig. S3 Molecular structure of some porphyrin dimer dyes.

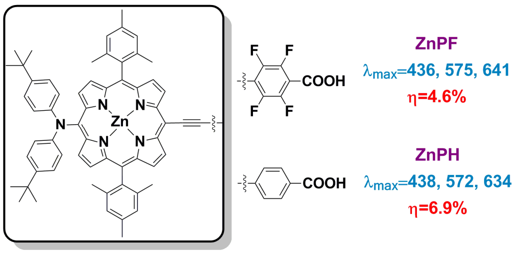

Fig. S4 Structure of ZnPF and ZnPH porphyrins.

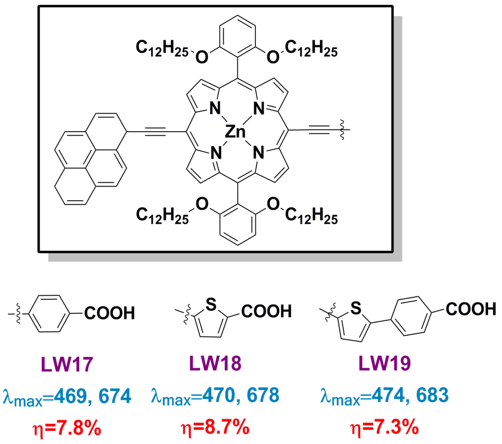

Fig. S5 Structure and performance of LW17-LW19 porphyrins.

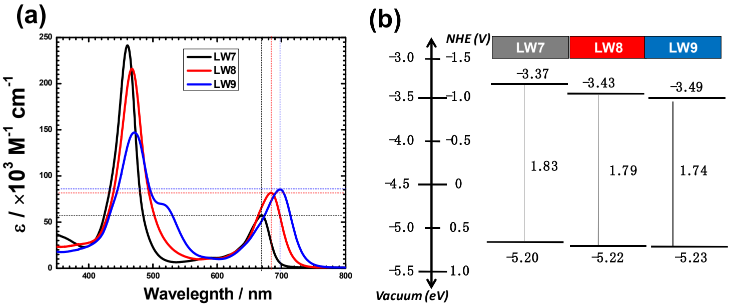

Fig. S6 (a) The absorption spectra of LW7, LW8 and LW9 porphyrin in THF solution; (b) the energy levels of LW7, LW8 and LW9 porphyrin.

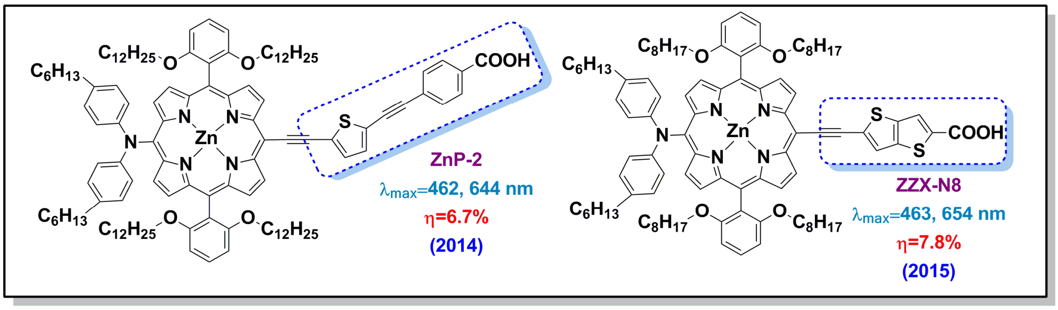

Fig. S7 Porphyrins featured with thiophene derivatives conjugated linker.

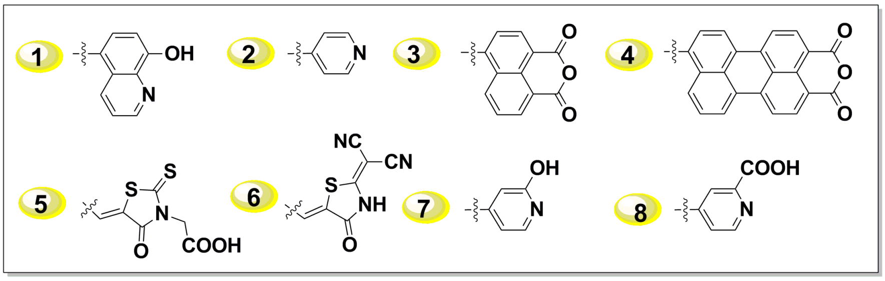

Fig. S8 Some 'un-typical' anchoring group porphyrin sensitizers.
